# *Listeria monocytogenes* and Listeriosis: The Global Enigma

**DOI:** 10.3390/foods14071266

**Published:** 2025-04-03

**Authors:** Christy E. Manyi-Loh, Ryk Lues

**Affiliations:** Centre for Applied Food Sustainability and Biotechnology, Central University of Technology, Bloemfontein X9301, South Africa; rlues@cut.ac.za

**Keywords:** *Listeria monocytogenes*, ready-to-eat foods, biofilm, antibiotic resistance, listeriosis, prevalence, control, global perspective

## Abstract

*Listeria monocytogenes* is an intracellular, Gram-positive, non-spore-forming, non-encapsulated, facultative anaerobic, rod-shaped, and psychrotrophic food-borne pathogen that causes the infection, listeriosis, thus it attracts great attention following listeriosis outbreaks, which are often associated with high mortality rates. The prevalence of listeriosis is quite low globally; however, the most recent and deadliest outbreak occurred in South Africa, during which 216 persons lost their lives. *L. monocytogenes* is endowed with the potential to multiply through a wide range of harsh environmental conditions, forming biofilms on varying surfaces in the food industry, as well as having persistent and antibiotic-resistant cells, which pose a major threat and burden to the ready-to-eat food industry. A more frustrating characteristic of this bacterium is its strain divergence, alongside an increased level of antibiotic resistance registered among the strains of *L. monocytogenes* recovered from food, humans, and environmental sources, especially to those antibiotics involved in the treatment of human listeriosis. Antibiotic resistance exerted by and among pathogenic food-borne microbes is an ongoing public health menace that continues to be an issue. Against this background, a thorough search into different databases using various search engines was performed, which led to the gathering of salient information that was organised, chronologically, based on *Listeria monocytogenes* and listeriosis. Altogether, the findings elaborated in this study present up-to date knowledge on different aspects of this pathogen which will improve our understanding of the mystery associated with it and the ways to prevent and control its dissemination through ready-to-eat foods. In addition, constant monitoring of the antibiotic resistance profiles of strains of *L. monocytogenes* from varying sources detected changes, giving an update on the trend in antibiotic resistance. Overall, monitoring of bacterial contamination serves as the key aspect in the control of the food safety output in the food industry.

## 1. Introduction

*L. monocytogenes*, alongside 20 others, is classified under the genus *Listeria* based on 16S ribosomal ribonucleic acid (rRNA), multilocus enzyme analysis, and deoxyribonucleic (DNA) information. Favourable growth conditions for the bacterium include temperatures ranging from –0.4 °C to 45 °C, low water activity, and a hydrogen ion concentration in the range of 4.4–9.6 [1]. These conditions impact the organism’s adaptability to growing and thriving under extreme conditions, as it occurs everywhere in nature (ubiquitous) and as a psychrotroph, thus posing primary threats to industries/companies dealing in food, because it is endowed with the potential to contaminate RTE foods [2]. As an overview of the *Listeria* species (*L. monocytogenes*), the genus *Listeria* comprises 20 species, which can be categorised further into non-pathogenic species, (including *L. thallandensis*, *L. costaricensis*, *L. goaensis*, *L. newyorkensis*, *L. booriae*, *L. riparia*, *L. grandensis*, *L. floridensis*, *L. cornellensis*, *L. aquatica*, *L. weihenstephanensis*, *L. fleischmannii*, *L. marthii*, *L. rocourtiae*, *L. ivanoii*, *L. seeligeri*, *L. innocua*, *L. grayi*, and *L. welshimeri*), and the main pathogenic species, *L. monoctyogenes* [3]. According to taxonomic hierarchy, these bacterial species are said to belong to the phylum *Firmicutes*, the class *Bacilli*, the order *Bacillales*, and the family *Listeriaceae*. They are described as rod-shaped, non-spore-forming, psychrotrophic, and facultative anaerobic bacteria that respond positively to Gram staining and calatase testing [4].

*Listeria* species can be found in a wide range of habitats, exhibiting motility at 30 °C, growing over a wide temperature range, and occurring as saprophytes. Also, they have the tendency to persist in the environment over long periods [5,6], especially in food-processing facilities due to environmental recontamination at the level of farms or plants. However, their detection in the food industry is considered as a marker for the presence, growth, and persistence of the notorious pathogen, *L. monocytogenes* [7]. Dufailu and colleagues [6] calculated an overall average prevalence rate of 23.7% *Listeria* spp. against 22.2% *L. monocytogenes* from the available reports in Africa. Moreover, *Listeria* species are widely distributed across the globe, but with varying prevalence rates in the different regions, countries, and continents of the world [6]. Amongst these species, only *L. monocytogenes* and *L. ivanoii* are known pathogens in this category. The former infects both humans and animals, whereas the latter infects just animals, mostly ruminants. This is the case because *L. ivanoii* harbours a *Listeria* pathogenicity island (LIPI 2) that encodes the enzymes, phosphocholinesterases that are essential for effective consumption of the phospholipids found in the erythrocytes contained in Ruminant blood, hence, this being the reason for the susceptibility of ruminants to *L. ivanoii* infections. Notwithstanding this, Allerberger [8] highlighted that the absence or presence of biochemical markers such as haemolysis and arylamidase can be employed to differentiate between the species *L. monocytogenes* and *L. ivanoii*.

More precisely, *L. monocytogenes* is an intracellular pathogen that is ubiquitous in nature, occurring in nearly every habitat, including the soil, water, dairy (cheese and milk), and meat products (ready-to-eat and raw), food-producing environments, and fresh vegetables, and dead/decaying vegetation, living as a saprophyte [5]. This bacterium, however, switches between saprophytic and host-associated lifestyles [3]. It is a pathogen associated with a great public and economic impact, causing listeriosis, which is acquired via the ingestion of RTE food and raw meat products harbouring this pathogen. Listeriosis caused by *L. monocytogenes* in humans can involve self-limiting gastroenteritis and an invasive and systemic disease; though its infections are rare (the bacterium is termed opportunistic), they are associated with huge mortality rates [9]. Evidently, this bacterium remains a major burden to food industries owing to its capacity to persist for years in food-processing/food-producing facilities. This is possible based on the unusual growth and survival traits of *L. monocytogenes* as well as the organism’s tendency to adhere to food contact surfaces, making it recalcitrant to the activity of the disinfectants/antimicrobials employed during sanitisation and cleaning processes designed to eliminate microbes in the environment. Altogether, this organism is set on a stage, where it is placed in the limelight, receiving constant concern as a public health challenge or threat [10].

Following the chronicle of information already published, a thorough search into different databases was performed using different search engines to gather salient information relating to *Listeria monocytogenes* and listeriosis. The search for published articles was performed employing the following descriptors: *Listeria monocytogenes*, “pathogenesis” “prevalence”, “transmission”, “RTE meat products”, “antimicrobial resistance”, and “listeriosis outbreaks”. This study therefore presents a comprehensive review of the up-to-date findings about *L. monocytogenes*, explaining the mystery associated with this bacterium registering lower prevalence rates of infection, but ultimately resulting in high mortality rates. In this light, vital knowledge or deep insights pooled from existing data are organised chronologically, cutting across the distinctive features, which include biofilm formation and tolerance to stressors (e.g., low temperature, acidity, and high water levels) by this organism, facilitating its survival under harsh environmental conditions (as it occurs everywhere). These conditions occur in food-processing environments, ready-to-eat foods, and animal-derived products, allowing the dissemination of *L. monocytogenes* via the food chain to human hosts, wherein, by reason of its suite of virulence traits and strain divergence, it can cause listeriosis (pathogenesis) or even an outbreak. Multidrug resistance demonstrated by the strains of this bacterium presents another area of great concern, since it can influence the outcome of the disease or infection. Several outbreaks of listeriosis were registered in different nations across the world with intense severity, but the deadliest outbreak occurred in South Africa, emphasising the need to understand the key factors for its epidemiology, both in developing and developed countries, as has been elaborated upon in this manuscript. Furthermore, ways to prevent and control *L. monocytogenes* are equally discussed as the public needs this knowledge to mitigate the spread of this pathogen. Altogether, the facts and materials assembled in this manuscript will help to improve the understanding of *L. monocytogenes* and listeriosis on a global scale.

## 2. Distinctive Features of *L. monocytogenes* Relevant for Survival in Varied Habitats

The outstanding ability of *L. monocytogenes* to adapt to stress conditions that occurs in varying environments is the reason for its ubiquitous nature. The bacterium possesses numerous unusual features that lead to difficulty in controlling the bacterium in foods, food-producing plants, or preparation; hence, permit its growth to levels that will cause infection/illness [11].

### 2.1. Biofilm Formation

Microorganisms exist either freely (planktonic forms) or in a community or consortium, relating the host. Notwithstanding this, biofilms are the primary means by which microorganisms live in nature. To withstand untoward environmental conditions and to cope with certain levels of known antibiotics, microbes tend to cover themselves with a protective layer. This is referred to as a biofilm [12]. A biofilm is defined as an ordered and arranged group of microbes (either of several species or the same species) living within an extracellular polymeric substance matrix (EPS) released by them, wherein they are attached to each other on living or non-living surfaces. The capability of microbes to form biofilms is viewed as an adaptable attribute of microbes; therefore, it is an age-old survival mechanism which provides the biofilm-producing bacterium with greater environmental stability, access to nutritional sources, productivity and interactions, tolerance to biocides, alongside a stronger ability to grow in an oligotrophic milieu unlike the planktonic cells [12,13]. *L. monocytogenes* relies on its rich catalogue of surface structures for biofilm formation [14]. The term surfactome is used in describing the entire band of molecules, which include cell wall proteins, teichoic acids, and peptidoglycan located at the surface of the cell wall of this bacterium [15]. Compared to their planktonic forms, these microorganisms occurring as biofilms demonstrate differences in terms of growth rate and expression of genes.

**Figure 1:**
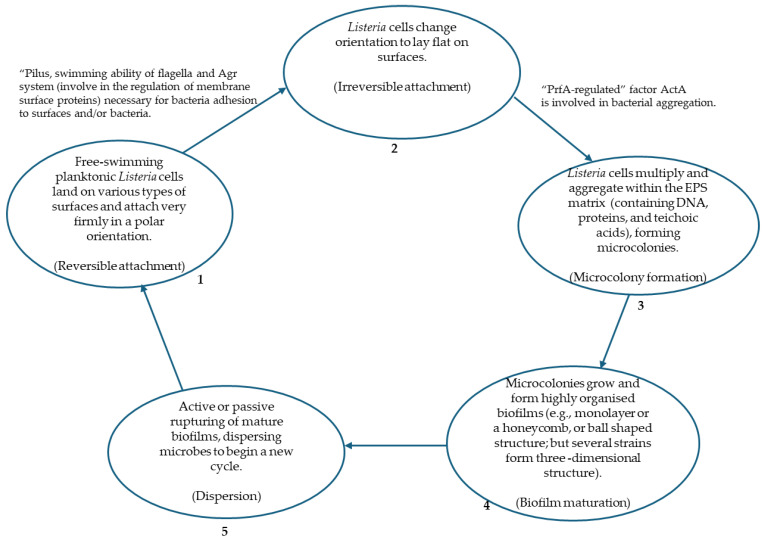
Life cycle of biofilm formation in *L. monocytogenes*.

The process of biofilm formation is termed complex and involves five steps as the microorganism transitions from a planktonic free-swimming organism to a biofilm-producing sessile form [12,16]. The different steps occur in sequence and involve attachment (reversible and irreversible), microcolony formation, maturation of biofilm, and dispersal [17], as shown in Figure 1. The activities of two signalling cascades (extracellular quorum sensing and the intracellular cyclic dinucleotide) are vital in this process, which begins with the loose and reversible attachment of planktonic microorganisms to surfaces in a polar orientation. The swimming ability of the flagella is very critical to bacterial attachment on both biotic and abiotic surfaces. In the attachment of *L. monocytogenes* cells to surfaces, genes associated with flagella synthesis and motility include *flaA*, *fliP*, *fliG*, *fliE*, *motA*, *motB*, *mogR*, and *degU* [18]. Subsequently, microorganisms change their orientation (from polar to flat) and become irreversibly attached to the surface, developing resistance to a host of physical factors that are meant to prevent the formation of biofilms [19]. Following the successful attachment of microbes to any surface, the organisms begin to multiply and aggregate within their self-produced EPS, resulting in the formation of a microcolony. In the maturation step, EPS performs crucial roles by helping in the attachment of microbes to surfaces, stabilising the three-dimensional structure of the biofilms, grouping cells together, as well as offering protection against various stresses [20].

A mature biofilm is made of three layers, which includes an inner regulating layer, the middle microbial basement layer and an outer layer occupied by planktonic microbes that are ready to exit the biofilm [21]. The final or last stage of this process involves the active (motility- and EPS-degradation-dependent dispersion) or passive (physical factors) rupture of the mature biofilm, dispersing microbes to begin a new cycle of biofilm formation. According to Soumya and coauthors [22], the dispersal stage symbolises the end of the previous biofilm formation cycle and the beginning of another or a new cycle. The lack of nutrients, stiff competition, and overgrown populations, added to variation in environmental factors, among others, are described as the major factors that are responsible for the dispersal of mature biofilms.

Biofilm formation can be described as a highly complex process that is highly regulated [19,23]. In *L. monocytogenes*, the quorum sensing system (Agr, system, comprising four genes, namely, *arg A*, *arg B*, *arg C*, *arg D*), virulence regulator, PrfA, and stress response regulator sig B play crucial roles in the development of biofilm [24]. Oliveira and colleagues [25] expressed that the process of formation is synonymous with an enterprise as the microbial community works together, pursuing a common goal. A biofilm constitutes primarily of microbial cells (10–25%) and an EPS matrix of between 75 and 90%; the EPS matrix comprises polysaccharides, extracellular proteins, extracellular DNA (eDNA), water, surfactants, and lipids. The microbial population contained within the EPS matrix of the biofilm can be of the same species (single) or different species (multiple) or a combination of varied microorganisms (bacteria, filamentous fungi, protozoa, algae, archaea, and yeast). The microorganisms demonstrate collective cooperation, source capturing, increased survival, or resistance against antimicrobials as characteristic features [20,26]. Variation occurs in the biofilm formation ability of the different strains of *Listeria monocytogenes*, wherein the strains with more rigorous extracellular polymer secretion and self-agglutination abilities display a more noticeable ability to form biofilms [27]. The entire process of biofilm formation depends on the interactions between the bacterial cells, the substrates, and the surrounding media [28]. However, the process is affected by a host of external factors, including pH, hydrodynamic forces, Brownian movements, gravitational forces, and secondary messengers among other signalling molecules. In addition, the structural characteristics of the organisms (microbial community composition), the nature of the surfaces (which is characteristic of the substrate surface on which the biofilm must be formed, e.g., wettability, porosity, and roughness), gene regulation, quorum sensing, the physicochemical properties of the substrate (hydrophilicity, hydrophobicity, surface energy, functional groups), as well as the environmental factors (medium characteristics, e.g., temperature, pH, flow rate of medium) have an influence on the biofilm formation process [23,29].

*L. monocytogenes* usually occurs in complex multi-species biofilm forms, facilitating its adaptation and survival in food-processing environments for months or even years [30]. Galie et al. [31] revealed that multi-biofilms were the major sources of contamination in the food industry. The multi-species biofilms usually present with properties, unlike the single-species biofilms. For instance, Chen and colleagues [32] remarked about a higher resistance to disinfectants and antibiotics of the mixed biofilms formed by *L. monocytogenes* with other bacteria. Notwithstanding this, the formation process of multi-species biofilms is the same as single-species biofilms, and *L. monocytogenes* is observed to form complex multi-species biofilms with other bacteria (including *S. aureus*, *Pseudomonas*, *E. coli*, *Bacillus cereus*, *Lactiplantibacillus plantarum*, etc., in different foods [33]. Temperature remains one of the main factors affecting the production of biofilms by *L. monocytogenes* and other bacteria. The dominance of a species in a multi-species biofilm is affected by temperature; the dominance of *L. monocytogenes* was observed in a multi-species biofilm developed at 30 °C. Low-temperature conditions within a range of temperatures could improve interactions between bacterial species, causing a tighter biofilm structure, yet an extremely low temperature will prevent biofilm formation. This was observed in the inability to form biofilm through the co-culturing of *L. monocytogenes* and *Enterococcus faecalis* at 7 °C, but a drastic increase in biomass of the multi-species biofilm culture was noted following a rise in temperature to 25 °C [34].

In multi-species biofilms, *L. monocytogenes* interacts either competitively or synergistically with the other bacterial communities. Sadiq and colleagues [35] described competitive interaction as a scenario or an interactive style in multi-species biofilms, where the different bacterial species or communities compete for limited nutrients/food supply, living space, and vital metabolic substances, or secrete metabolites that inhibit the growth of other microorganisms. As a matter of fact, bacteria display differences in colonisation times which will in turn affect their distribution; additionally, some bacteria secrete antagonistic metabolites in a multi-species biofilm. Accordingly, the findings of Shao and coauthors [36] revealed that biofilm formation by *L. monocytogenes* was significantly inhibited by a large molecule (>30 kDa) secreted by *Leuconostoc mesenteroides* W51. Also, De Grandi and colleagues [37] highlighted the preferential colonisation of the surfaces on stainless steel as the organism competed for survival space with *L. monocytogenes*. Thus, decreasing the proportion of *L. monocytogenes* in the multi-species biofilms. These interactions can be affected by the type of food substrate (whey protein, skimmed milk, etc.), the state of the culture system (static or continuous flow conditions), added to other external factors [38]. The interactions can ultimately influence the development, structure, function, and resistance of multi-species biofilms. Chen and colleagues [32] opined that regulating the external factors that affect the growth of multi-species biofilms may represent one of the approaches to resist cross-contamination in the industry by *L. monocytogenes*.

The surface of the substrate could be plastic, steel, or titanium, among others, but a surface that supplies both moisture and nutrients is said to be an ideal environment for the formation of biofilms [21]. Lee and colleagues [39] in their study revealed that distinct external factors exerted effects on specific steps of biofilm formation; abrupt deprivation of nutrients led to greater cellular adhesion, whilst prolonged deficiency of nutrients hindered the maturation of biofilms, and the addition of salt resulted in an increased biofilm production. The biofilm can have either anionic, cationic, or neutral properties; the anionic tendency permits the divalent cations to associate with the inter-link strands of the polymer, providing a more binding force to the mature biofilm [40]. However, the different charges and ions offer structural integrity to the EPS of the biofilm, causing the biofilm to develop the ability to survive in environments of extreme shearing forces [20]. Just as this bacterium shows great diversity in its strains, *L. monocytogenes* strains equally differ in their ability to produce biofilms. Accordingly, Takahashi and colleagues [41] noted that among the *L. monocytogenes* strains (71) employed in their study, which were recovered from food, there existed a marked correlation between the isolates belonging to the lineage I (1/2b, 4b) and biofilm formation, as opposed to the isolates grouped under lineage II (serotypes 1/2a, 1/2c). Contrarily, Borucki and colleagues [42] remarked about the higher capacity of biofilm formation by *L. monocytogenes* strains categorised under lineage II. Moreover, Carpentier and Chassaing [43] highlighted the varying degrees of biofilm production by isolates belonging to the same clonal lineage, suggesting that environmental factors could have an influence on the process. Therefore, based on their biofilm-forming ability, the different strains of this bacterium can be classified as weak, moderate, or strong biofilm producers [44]. Osek and colleagues [45] ascribed the discrepancies in the findings of the above-mentioned researchers to the different methods that were applied in the different studies, along with several strains. However, through whole genome sequencing technique, Keeney and coauthors [46] suggested that serotype-specific differences in biofilm formation could be associated with the occurrence of stress survival islets I (SSI-I) based on analysing the information gathered from environmental and food-derived isolates of *L. monocytogenes* (166 strains).

*L. monocytogenes* has demonstrated its capacity to adhere to and develop on several materials including polystyrene, polytetrafluoroethylene, stainless steel, polyester, and rubber, that are commonly used in food facilities and premises [42,47]. Notwithstanding this, the affinity displayed by the bacterium to the above-mentioned materials varies greatly, depending on the different types [48]. This may suggest that the properties of the surface material have a significant effect on the development of biofilms by *Listeria*; for example, topography [49]. In this light, non-smooth or rough surfaces, precisely, worn or corroded surfaces provide greater opportunities for biofilm formation due to defects or crevices on their surfaces, capable of trapping nutrients and water necessary for the proliferation of bacterial cells [50]. These crevices might also shield or shelter the bacterial cells from the actions of cleaning and disinfection agents [51]. For this reason, stainless steel is employed as an ideal material in the manufacturing of most equipment and utensils that are utilised during food processing. The hardness of the material, its resistance to corrosion, added to its ability to be polished, help in ensuring that the surface remains smooth [52]. Clearly, Soares et al. [53] remarked on the greater ability of bacterial cells to quickly adhere to polystyrene surfaces rather than hydrophilic counterparts, e.g., glass or stainless steel. This is attributed to the hydrophobic nature of the polystyrene surface, which is also true for nonpolar surfaces (plastics).

Silva-Dias et al. [54] affirmed that hydrophobicity encourages biofilm formation via improving microbial adhesion, therefore heightening the antibiotic resistance of cell communities as well as their virulence. Interestingly, contradictory findings were reported by others who registered a higher rate of biofilm formation on hydrophilic surfaces as opposed to hydrophobic surfaces. These conflicting results are possible, as discrepancies are inevitable due to the different experimental designs and the test strains that were implemented in the different studies. In addition, temperature, pH, sodium chloride and the availability of nutrients are also counted among the specific factors necessary for biofilm development by *L. monocytogenes* [44]. Of great consideration is the interactions between *L. monocytogenes* and other microorganisms. This can equally inhibit or promote the organisms’ biofilm development based on the strain or available conditions.

#### 2.1.1. Implications of Biofilm Formation

##### Antibiotic Tolerance/Resistance

*L. monocytogenes*, as a bacterial species, deploys quite a few defence approaches to dodge the deleterious effects of lethal treatments, including antibiotic therapy. The terminology “resistance” is used to illustrate the potential of a bacterium to grow at higher antibiotic concentrations, not considering the time of exposure to the treatment [55]. Tolerance and persistence, on the other hand, are two other terms employed in describing the defence strategies of a bacterium. They refer to the capacity of *L. monocytogenes* to survive temporary bactericidal therapy for a longer period, regardless of the fact that the minimum antibiotic concentration required to terminate their growth remains unchanged [56]. Brauner and colleagues [56] explained that antimicrobial resistance is determined by measuring the minimum inhibitory concentration (MIC), whereas antimicrobial tolerance is determined by measuring the time needed to obtain a 2log reduction in the microbial population. Although both tolerance and persistence can be expressed through similar principles or mechanisms, the former is associated with the entire bacterial population while the latter is simply linked to a subpopulation of bacterial cells [57]. The most remarkable feature of the persistent bacteria is the presentation of a biphasic killing curve that describes the presence of one initial major subpopulation that is highly sensitive to the treatment, and one minor subpopulation that demonstrates a greater resistance to the treatment. Thus, persistent bacteria are categorised as a subpopulation of microbes that, when subjected to a bactericidal treatment are killed at a slower rate, unlike the remainder of the population among which they are found [55].

In addition, persistent bacteria can withstand treatments that are lethal longer than their counterpart bacterial cells because they are able to transform into transient non-growing cells, generated either following stressful stimuli (termed Type I persistence) or in the absence of external signals (termed Type II persistence) [58]. Nalbone et al. [55] reported that *L. monocytogenes* ATCC 7644 formed persistent cells that could survive mild heat for a longer duration after being exposed to the salt concentration that supported its growth. It is noteworthy that the lag phase of the persistent cells was extended immediately, following the removal of the stress stimuli, and the growth was resumed in a fresh culture medium. According to Lakicevic and colleagues [59], persister cells build up pools of cells that can cause contamination, therefore increasing the likelihood of infections in humans. Moreover, Al-Nabulsi and coauthors [60] revealed that environmental stress could trigger resistance in *L. monocytogenes* against a broad spectrum of antibiotics. This further explains the increased minimum inhibitory concentration (MIC) of chosen antibiotics since the bacterium is exposed to selected stress conditions that usually occur in the food-processing environment. Relatedly, Wiktorczyk-Kapischke et al. [61] remarked that the organism’s capacity to form biofilms and the minimum inhibitory concentration (MIC) values of the antibacterials can be altered by environmental stress. Furthermore, the degree of expression of selected genes can equally be affected, thereby increasing the survival and virulence of *L. monocytogenes*. Also, the structure of the biofilm, the shape of the matrix, and the 3-D arrangement of the microbes are said to exert effects on antibiotic resistance and other functional features of a biofilm [62]. The presence of other microorganisms alongside *L. monocytogenes* in a food-processing environment can provoke the development of robust multi-species biofilms, which most probably could enhance the organism’s tolerance to sanitisers, thus improving its survival and persistence [63]. The tolerance of biofilms to antimicrobials or sanitisers can take place via several mechanisms, including (i) a decreased diffusion of the antibiotics into the biofilm structure, (ii) a degradation of the antimicrobial molecule into non-inhibitory compounds, (iii) a transition into a nearly dormant state termed persister cell physiology [64].

Furthermore, the occurrence of antibiotic-resistant strains of bacteria, including *L. monocytogenes*, is a dire global public health concern [4,48,65]. The treatment of listeriosis in humans, which is the eradication of *L. monocytogenes* in a bid to ensure healing in the individual, involves the use of antibiotics, including gentamicin, penicillin, amoxicillin/ampicillin, tetracycline, chloramphenicol, rifampicin or trimethoprim, and sulfamethoxazole, either as a single or a combined therapy [66,67]. Recently, a combination therapy consisting of ampicillin or amoxicillin with gentamicin is the primary regimen employed in cases of human listeriosis [68]. However, selecting the proper antibiotics with the required bactericidal action against *L. monocytogenes* necessitates the monitoring and the distribution of antibiotic resistance patterns and genes amongst *Listeria* species occurring in a certain region [4,69]. Clearly, antibiotic resistance is a natural phenomenon inherent to the use of antibiotics, implying when antibiotics are in use over an extensive period, there is a high chance or likelihood of the development of antibiotic resistance [70]. Antibiotic resistance describes the capacity of the bacterium to survive or thrive or grow in the presence of antibiotics at concentrations that are employed in clinical practice, whereby the microbe changes its response to the activity of the antibiotics [71].

The phenomenon of antimicrobial resistance has been evolving over time owing to the involvement of plasmids, transposons, and integrons (termed mobilisable genetic elements, MGEs), which facilitate the transfer of genes horizontally [72]. This may occur either by mutation or via the acquisition of resistance factors that are contained in mobile genetic elements, which are usually accelerated by biofilm formation [73]. It has been observed that *L. monocytogenes* acquires antibiotic resistance via efflux pumps, biofilm formation, and the exchange of antibiotic resistance determinants with other species via horizontal gene transfer (adaptive mechanisms) [73]. According to Ruiz-Bolivar and colleagues [74], *Enterococcus* and *Staphylococcus* species are the common sources of resistance genes for transmission to *L. monocytogenes*. Precisely, acquired resistances in *L. monocytogenes* were expressed through acquiring resistance genes that encode ribosomal protection proteins (*tetM/tetS*), efflux proteins (*tetK/tetL*) conferring resistance to tetracycline, alternative dihydrofolate reductases (dfrD/dfrG) that evade the bacteriostatic effect of trimethoprim, 23S ribosomal RNA methyltransferases (*ermB*) that shield erythromycin from binding, and chloramphenicol acetyltranferases (*catA)* that inactivate chloramphenicol by the addition of an acyl group [75]. The authors further registered the acquired antimicrobial phenotypes of *L. monocytogenes* to include tetracyclines (primarily because of *tetM*), trimethoprim (*dfrD*), lincosamides (*lnuG*), macrolides (*ermB*, *mphB*), and phenicols (*fexA*). Furthermore, core genes in *L. monocytogenes* can be mutated, leading to a decrease in the affinity of the drugs for their enzymatic target sites, indicating resistance to rifampicin (*rpo*) or streptomycin (*rm*) or ciprofloxacin (*gyrAB*/*parC*/*lde* mutations) [76].

Antibiotic resistance is an expanding phenomenon in many pathogenic bacteria, including *L. monocytogenes* [77]. Multidrug resistance of bacteria is now emerging worldwide and the detection of multidrug-resistant *L. monocytogenes* in food is considered to be a threat to public health globally [9]. This has undoubtedly rendered the treatment of listeriosis complex. Several studies conducted across different regions or countries of the world have registered varying percentages of antibiotic resistance or multidrug resistance in the said bacterium (Table 1). Accordingly, the antibiotic resistance demonstrated in *L. monocytogenes* can be expressed via resistance genes, including *fosX*, *lin*, *abc-f*, and tet(M), which were identified as the four most prevalent antimicrobial genes in the bacterium [78]. This observation is established owing to the emergence of resistance patterns continually over the years and from varying sources [79]. These discrepancies are attributed to the employment of several antimicrobials in various geographical regions at different times [4,80]. It is somewhat clear that the antibiotic resistance profile of a bacterium may be influenced by its strain, time, and season as well as the geographical origin (location or place) [81].

The global increase in the total demand for meat and its products has led to the adoption of intensive farming, necessitating the use of huge quantities of antibiotics to treat infections in animals as well as to increase productivity [82]. Notwithstanding this, antibiotics can be used in animal farming for other reasons, including growth promotion, prophylaxis/treatment, as well as metaphylaxis [82,83]. The extensive application of antibiotics in veterinary medicine or animal farming may provoke the emergence and the distribution of antibiotic-resistant genes via horizontal gene transfer within and between bacterial species in the environment [84]. Although most strains of *L. monocytogenes* demonstrate natural resistance against the third and fourth generations of cephalosporins and fluoroquinolones that are currently in use, there exists an observed variation in the level of resistance between the strains. These inconsistencies could be ascribed to the differences in antimicrobial uses both in humans and animals as well as geographical differences [85]. Accordingly, several studies noted different prevalence rates of resistance to varying antibiotics, including nalidixic acid, nitrofurantoin, tetracycline, rifampicin, fluoroquinolones, vancomycin, amoxicillin, and oxacillin, among others. However, the resistance demonstrated to the previously mentioned antibiotics by this organism is a cause for concern because these antibiotics are listed amongst the substances used in the treatment of human listeriosis, with fluoroquinolone and rifampicin being used frequently [86]. The observed prevalence rates of resistance to the said antibiotics could be correlated with the ease of availability, accessibility of the drugs, more common prescription, and relatively inexpensive price of the drugs employed by the local community to combat infections in the veterinary and the public health sectors [87].

Apparently, the bacterium exhibits the tendency to form biofilms on food, instruments, working surfaces, and utensils in food-producing facilities or environments to resist standard cleaning procedures, added to the application of disinfectants (antimicrobials) for sanitisation purposes [88]. Employing antimicrobials at low dosages or incomplete antimicrobial therapeutic regimens are the major factors explaining the origin and spread of antimicrobial drug resistance. However, the formation of biofilms by *L. monocytogenes* on various materials alongside the deficiency in the development of new antimicrobial drugs can go a long way to initiating resistance in the organism to antimicrobial activity [78,89]. Furthermore, antibiotic resistance is worsened by the use and misuse of antimicrobials at the level of the farm for several reasons stated elsewhere by different authors, in conjunction with the use of antimicrobials in subtherapeutic dosages for disinfection at the level of sanitisation in the food industries. These practices result in increased resistance among the bacteria that occur in the habitat, creating reservoirs, therefore increasing the opportunity for the spread of resistance to humans via food consumption. Several authors have emphasised the dilemma associated with antibiotic-resistant bacterial pathogens transferred to humans, whether directly or indirectly, causing serious and difficult-to-treat infections, which in turn can lead to life-threatening situations [84].

**Table 1:** Different virulence and antimicrobial resistance traits identified in *L. monocytogenes* isolates recovered from RTE food.

Description of Tested Samples	Countries	Prevalence Rates (%) of Antibiotic-Resistant *L. monocytogenes*	Antibiotics to Which Isolates Are Resistant	Genes Conferring Resistance and Virulence Genes	References
RTE foods (cooked red meat, cooked chicken, seafood, vegetarian, and baked egg products, raw lettuce, fruit salad, vegetable salad, dairy products, mayonnaise-based salad, deli salad, and desserts with milk) (Total samples = 201)	Turkey (Ankara)	35.3–94.1	Oxacillin, kanamycin, levofloxacin, teicoplanin, amoxicillin, rifampicin, ciprofloxacin 100% MDR	Not completed Virulence genes: *hly* A Listeriolysin	Sanlibaba et al. [90]
Artisanal foods (minimal and moderate processing) (Total samples = 400)	Chile	12.5–25	Ampicillin, trimethoprim-sulfamethoxazole	Not completed Virulence genes: *hyl*A, *prf*A, *inl*A	Bustamante et al. [91]
Retail foods (RTE foods and raw foods) Total samples = 3354)	China (Zhejiang province)	11.0	Tetracycline	Not completed Virulence genes: *prfA*, *hlyA*, *plcA*, *plcB*, *mpl*, *actA*, genes, *LIPI*-1, *inlA*, *inlB*, *inlC*, *inlJ*, *LIPI*-2, *LIPI*-3, *LIPI*-4	Zhang et al. [92]
Cake, raw meat, ice cream, minced beef, fish, unpasteurized milk, pizza (Total = 384)	Ethiopia (Gondar town)	16.6–66.7	Penicillin, nalidixic acid, tetracycline, chloramphenicol 16.7% MDR	Not completed	Garedew et al. [87]
Bovine milk (Total samples = 161)	China (Yunnan Province)	12.5–100	Ampicillin, tetracycline, trimethoprim-sulfamethoxazole, erythromycin, vancomycin, ciprofloxacin, meropenem 75% MDR	*novA*, *kdp*E, *NmcR*, *drfG*, *facT*, *norB*, *fusA*, *van RM*, *sul4*, *tetA*, *tetB*, *tetD*, *tetM*, *tetS* (amongst others, 99) Virulence genes (83): *actA*, *hlyA*, *inlA*, *inlB*, *inlC*, *inlJ*, *mpl*, *plcA*, *plcB*, *prfA*, *fliE*, *flgK*, etc.	Su et al. [93]
RTE foods including salads (lettuce, carrot, cabbage, sweetcorn, mayonnaise as options), meat pies (potatoes, minced meat, carrots as options), fried snails, and edible worms (Total samples = 411)	Southern Nigeria	47.77–100	Amoxicillin, cloxacillin, Augmentin, ceftazidime	Not completed Virulence genes: *hlyA*, *inlA*, *iap*	Ebakota et al. [94]
Raw fish, open-air market environment (Total samples = 862) Caesar salad, Olivier salad, burger, schnitzel, sushi, sausage (Total samples = 110)	Iran Iran (Tehran)	16.3–27.9 0–100	Tetracycline, ampicillin, cephalothin, penicillin, streptomycin Oxacillin, streptomycin, cotrimoxazole, clindamycin, cefoxitin, erythromycin	*TetA*, *tetM*, *ampC*, *penA* Virulence genes: *inlA*, *inlB*, *inlC*, *inlJ*, *actA*, *hlyA*, *iap*, *plcA*, *prfA*. *ermA*, *ermB*, *cfxA*, *mecA* Virulence genes: *hlyA* and *prfA*	Jamali et al. [9] Mirzaei et al. [95]
Milk samples (Total samples = 65)	South Africa (Eastern Cape)	42.86–71.43	Sulfamethoxazole, trimethoprim, erythromycin, cefotetan, oxytetracycline 85.71% MDR	bla_TEM_, bla_SHV_, bla_Z_, *tetA*, *tetD*, *tetG*, *tetM*, *tetK*, *aph(3)-IIa (aph12*)^a,^ *sul2*, *sul1* Virulence genes: *prfA*, *plcA*, *plcB*, *inlA*, *inlC*, *hylA*, *mpl*, *actA*, *inlJ*, *inB*	Kayode and Okoh [96]
Poultry meat (Total samples = 250)	Egypt (Mansoura city)	58.33–91.67	Tetracycline, oxytetracycline, penicillin, amoxicillin, augmentin, and ceftazidime 79.2% MDR, 16.7% XDR	Not completed Virulence genes: hlyA, actA, iap	Zakaria and Sebala [97]
Beef and chicken (Total samples = 90)	Iran (Zanjan city)	91.7–100	Trimethoprim-sulfamethoxazole, tetracycline, penicillin, and gentamycin.	Not completed Virulence genes: *hlyA*	Farhoumand et al. [98]
Retail RTE foods (cheese, cooked meats, pre-processed fruits and vegetables, mixed dishes with raw and/or cooked ingredients) (Total samples = 436)	Chile (Maule region)	21.43–100	Ampicillin, tetracycline	*fosX*, *lin*, *norB*, *mprF*, *tetA*, *tetC* Virulence genes: *hlyA*, *prfA*, *inlA*,	Parra-Flores et al. [99]
RTE products: Processed dairy, bovine meat, and poultry products. Processed pork meat (sausage, ham and bacon) and fish products (Total samples = 8151)	Romania (North-Western region)	23.07–26.92	Oxacillin, trimethoprim-sulfamethoxazole, penicillin, tetracycline 23.07% MDR	*tetC*, *tetM*, *tetK*, *ampC*, *drfD* Virulence genes: *hlyA*, *prfA*	Duma et al. [100]
Meat, seafood, dairy, confectionary products, sauces, RTE dishes, food-processing environment (Total = 269)	Italy (Lazio region)	78.44–88.48	Oxacillin, fosfomycin, flumenique 87.36% MDR	Not completed	Rippa et al. [101]
Processed raw meat products (Total samples = 270)	Jordan (Amman)	5–56.6	Neomycin, tetracycline, kanamycin, erythromycin	Not completed	Al-Nabulsi et al. [60]
Locally processed fermented foods, e.g., garri, Qunu, zobo (Total samples = 80)	Nigeria (Ethiope, Delta State)	62.5–100	Penicillin, clindamycin	Not completed	Beshiru and Uwhuba [102]
RTE foods (Total samples = 105)	India (Tamil Nadu)	24–52	Methicillin, clindamycin, lincomycin, azithromycin, carbenicillin, amoxicillin	Not completed	Elavarasi et al. [103]
Raw kebab and hamburger (Total samples = 100)	Iran	66.7–100	Amoxicillin, penicillin, cefalexin	Mec A Virulence genes: Not completed	Rajei et al. [104]
Raw meat (Total samples = 190)	Turkey (Ankara)	86.90–100	Ampicillin, fosmycin, nalidixic acid, linezolid, clindamycin, piperacillin. 73.91% MDR	Not completed Virulence genes: hlyA	Sanlibaba et al. [105]
Raw milk, ice cream, minced meat, fish fillet, sausage (Total samples = 250)	Egypt (Menoufiya governorate)	41.2–76.4	Oxytetracycline. Trimethoprim-sulfamethoxazole, chloramphenicol, doxycycline, levofloxacin, azithromycin 100% MDR	Not completed Virulence genes: hlyA, iap, actA	Abdeen et al. [106]
RTE foods including meat-free cig kofte, kavurma, pastrami, doner, salad, dessert, cheese, ice cream (Total samples = 300)	Turkey	46.7–80	Fusidic acid, ceftriaxone, clindamycin 40% MDR	Not completed Virulence genes: *actA*, *iap*, *inlA*, *inlB*, *inlC*, inlJ, plcA, prfA	Arslan and Özdemir [107]
Retailed beef and beef products (Total samples = 400)	South Africa (Gauteng Province)	62.5–100	Clindamycin, penicillin, nalidixic, cefotaxime 75.7% MDR	Not completed	Gana et al. [108]

Taking into consideration Table 1, the information/data gathered revealed the contamination status observed, as well as the varying prevalence of resistant and multidrug-resistant isolates of the bacterium *L. monocytogenes* in several food sources investigated in different regions and countries. The discrepancies in the prevalence rate of *L. monocytogenes* recovered from the different foods and countries could be ascribed to the types of antibiotics used, the methods employed in the different studies, and the expertise of the investigators, added to the time/season during which the study was performed [84,109]. Globally, the proportion of antibiotic-resistant strains varied from the low- and middle-income countries (developing) to the developed countries. However, a high level of resistance is presented by *L. monocytogenes* to commonly used antibiotics (including amoxicillin, tetracycline, trimethoprim-sulfamethoxazole, penicillin, ampicillin, erythromycin, etc.) in the African countries. A nation’s economy, in addition to the level of national income, has a huge impact on the making and implementation of policies that govern waste management (wastewater, wastewater treatment plants, municipal and domestic wastes, etc.), hygiene and sanitation, water, the purchase and use of antimicrobials both in the clinical and animal farming settings, in addition to other public services. Larsson and Flach [110] remarked that all antibiotics used consistently for the same or varying purposes on a consistent basis will eventually end up in the environment, further heightening the pace of development of antimicrobial resistance. Consequently, identifying the high-risk environments that are responsible for the evolution and spread of antimicrobial resistance is imperative, and it presents as a primary step in global strategies to mitigate antimicrobial resistance [111]. Effective management of waste facilitated by adequate or high income and good governance/surveillance of a nation, in addition to the reduction in the release of antimicrobial residues in the environment, can effectively limit the dissemination of antibiotic-resistant bacteria and their resistance genes, thus controlling the hazards associated with antimicrobial resistance [112]. In addition, Ahmad and colleagues [111] highlighted that the chances of transmitting antimicrobial resistance can be influenced by the standards of control of infection, sanitation, accessibility to clean water, accessibility to assured quality antimicrobials and diagnostics, and travel, added to migration.

Antimicrobial stewardship is another key factor that can influence the varying levels of antibiotic resistance observed across the nations of the world [113,114]. This programme, whether implemented in clinical settings or in animal settings, will determine the overall quantity of drugs in circulation or in use in humans and animals. The enforcement of laws governing the purchase and use of these drugs as well as diligence in the implementation of antimicrobial stewardship in both the clinical and animal settings may vary from one nation to the other. The misuse and overuse of antibiotics increases the levels of antibiotics in the environment overwhelmingly, as well as the rate of their spread [115,116]. This tendency, though it is a common global phenomenon, tends to vary from nation to nation, affecting the level of antibiotic resistance. In addition, Tadesse et al. [117] ascribed the varying prevalence rates of this pathogen among different countries to the lack of consistency in the measurement and reporting of susceptibility data by different laboratories in different countries and, sometimes, even within a particular country. To this effect, high-income countries (i.e., laboratories in Europe using the EUCAST system) have employed harmonisation efforts, but African countries also need to employ similar harmonisation efforts based on standardising antimicrobial resistance methods and interpretation guidelines. This is so that better comparison between findings from different laboratories and countries can be reached and resistance tracking can be improved.

Moreover, on a national scale, regional variation in the percentage of antibiotic-resistant strains was observed within countries (based on our data), including South Africa, Chile, Iran, Turkey, Nigeria, and China. For example, the discrepancies in the percentages of antibiotic resistance in animal products (meat and milk) between the studies performed in Gauteng province (Johannesburg, city) and Eastern Cape Province (Alice, location) in South Africa is not surprising. Manyi-Loh and Lues [84] stated that the socioeconomic and political factors and strain divergence in *L. monocytogenes*, the geographical area, time, and climatic factors, could encourage discrepancies in the level of antibiotic resistance exhibited by this pathogen across the different provinces. Nevertheless, the observed differences in the level of antibiotic resistance in *L. monocytogenes* isolates in South Africa over the investigated period could indicate a rising pattern in antimicrobial resistance in this opportunistic pathogen.

In relation to the resistance activity expressed by the bacterial isolates, the different terms multidrug (MDR), extensivelydrug (XDR), and pan-drug resistance (PDR) are used to describe the magnitude or level of the effect [118]. According to Table 1, the lowest percentage of MDR (16.7%) was registered in Ethiopia, thus suggesting that this fraction among the bacterial test isolates demonstrated non-susceptibility to at least one agent in three or more antimicrobial classes [119], while Zakaria and Sebala [97] noted a fraction (16.7%) of their tested isolates that were extensively drug-resistant in their study performed in Egypt. Being extensively drug-resistant suggests that the bacterial isolates displayed resistance to at least one antibiotic among all the classes of antibiotics tested, i.e., they maintained their sensitivity to one or two classes. Although, the condition becomes more crucial as we traverse from MDR to XDR, both the MDR and XDR bacterial isolates identified and recovered among food-borne microbes are a cause for great concern, presenting critical threats to public health because the bacteria can now grow in the presence of chemicals (drugs) that would normally destroy or inhibit them [120]. Consequently, antibiotics, when employed in the treatment of infections caused by these bacterial isolates, will fail to produce therapeutic effects—a situation that has been associated with several mishaps as presented elsewhere by several authors [84,121]

Overall, it is obvious that recent data on the antimicrobial resistance of *L. monocytogenes* found in RTE food are not available in most countries of the world. This is a notable finding because Tadesse and colleagues [117] mentioned that a vital step towards designing targeted strategies to address the global antimicrobial crisis involves the availability of routine and research data on the susceptibilities of the different bacterial pathogens of public health interest. Notwithstanding this, the prevalence rates of antibiotic-resistant strains can lead to a reduction in production, which might affect livelihoods, including food security. Notwithstanding this, owing to the rise in global trade and travel, antimicrobial resistance could be disseminated between regions, countries, and continents [122], thus suggesting a huge public health threat to the hypersensitive consumer populations [99]. Apparently, understanding and knowledge is revealed, facilitating the adoption of appropriate measures to avoid the contamination and transmission of resistant bacteria by way of food [101]. It is therefore necessary to seek ways to curb contamination, as the data recovered indicate the possibility of listeriosis outbreaks or provocation of human listeriosis.

Moreover, as indicated in the table, microbiological data are of serious concern; therefore, close monitoring of MDR and XDR in *L. monocytogenes* is paramount in the different regions of a particular country as well as in the different nations of the world (i.e., global and national actions) when evaluating the microbiological quality of RTE foods and foods derived from animal origin. Prestinaci and colleagues [123] mentioned that the World Health Organisation recognised the necessity for a better and coordinated global effort to contain antimicrobial resistance through slowing the emergence and reducing the dissemination of antimicrobial-resistant microbes. This involves individuals, health professionals, the agricultural sectors, policymakers, and the healthcare sectors [124]. This will lead to the adoption of effective measures that will decrease the threat of antimicrobial resistance within a country and around the world [125].

The antimicrobial fitness of the bacterium could be expanded via acquiring resistance traits/genes from the *Enterococcus* and *Streptococcus* species [126]. Consequently, the =in vitro assessment of the antimicrobial agents prior to treatment becomes inevitable, as does searching for novel and alternative antimicrobial agents. Exercising delays in treatment, added to the wide dissemination of multidrug-resistant *L. monocytogenes* harbouring a suite of virulence traits, are regarded as the major triggers for the manifestation of the infection [127]. Therefore, emphasising the ongoing surveillance of *L. monocytogenes* in different food types, as well as the significance of detailed monitoring of antimicrobial resistance to guide the development of sustainable strategies by public health practitioners to reduce contamination (prevalence and antimicrobial resistance) and subsequent infection [100]. Overall, the data provoke provide an urgent and timely investigation into the pathogenicity, virulence, and antimicrobial resistance of the pathogen worldwide on a continuous basis to identify or detect novel resistance genes [114].

In addition, antibiotic resistance may equally be related to increased virulence or transmission, which may become crucial in the global spread and dominance of selected resistant bacteria, pending the type of plasmid [128]. Although virulence and antibiotic resistance might have evolved over different timescales, these two phenomena display similar characteristics, including both processes which are vital to the survival of a bacterium when subjected to hostile conditions. Also, their determinants are disseminated between species or genera via horizontal gene transfer, directly involved with efflux pumps, porins, alterations in cell walls, as well as their association with infection. However, antibiotic resistance is said to increase the virulence or fitness of a bacterium by aiding it to colonise new niches. It is explained that antibiotic resistance would enable a bacterium to colonise niches where other bacterial species could not and would even be capable of displacing the commensal flora in certain ecological niches (e.g., in niches with high antibiotic pressure). Antibiotic resistance is not in itself a virulence factor but can provoke the development of an infection in specific situations (e.g., hospital) [129].

*L. monocytogenes* has well-known important determinants necessary for its pathogenicity, and these include actin, internalin, listeriolysin, invasion-associated protein, phosphatidylinositol phospholipase, and virulence regulator [130]. However, the coexistence of the virulence traits with antibiotic resistance is quite worrisome because it has negative effects both at the clinical and environmental levels—that is, it promotes clinical outbreaks that could result in life-threatening infections, posing serious risks to public health [131]. According to Schroeder and colleagues [73], the advent of antibiotic resistance and increased virulence often occur almost simultaneously. Therefore, identifying the major genetic interactions between these two in a biofilm bacterium is necessary, leading to the identification of novel drug targets, which in turn will eventually provide a drug discovery and development pathway to improve treatment options for chronic and recurring bacterial infections [73]. In recent times, multidrug resistance has been detected in *L. monocytogenes* isolates recovered from varying sample sources, including animals, different food types, and human cases of listeriosis [132]. To be precise, the rising prevalence of antibiotic resistance in *L. monocytogenes* strains was very notable, recovering them from foodstuffs. It must be pointed out that the growth in antibiotic resistance in this bacterium could be attributed to the progressive acquisition of mobile genetic elements, including plasmids and transposons from cells of various genera of bacterial origin [71]. The biofilm serves as a pool harbouring genes that are resistant to antibiotics (the resistome), promoting the spread of antibiotic resistance genes to other pathogens via the process of horizontal or lateral gene transfer, occurring as conjugation, transformation, and transduction [133]. Summarily, Olaimat and colleagues [71] emphasised that some *L. monocytogenes* strains recovered from foodstuffs presented with intrinsic resistance to various antibiotics. The widespread use of antibiotics, the use of sub-inhibitory levels of antibiotics, horizontal gene transfer, the presence of persister cells, exposure to environmental stressors, and biofilm formation are the factors playing vital roles in the emergence of antibiotic resistance in the bacteria occurring in food-processing chains and the environment.

*L. monocytogenes* is a known biofilm-producing bacterium; therefore, several possibilities could explain the cause of antibiotic resistance in the bacterium. In addition to horizontal gene transfer of antibiotic resistance genes, the slower growth rate of the biofilm-associated bacterium causes a slower uptake of the antimicrobial agents, resulting in a suboptimal intracellular drug concentration that cannot trigger any bactericidal effect, leading to the killing of the bacterium [20]. Mirghani and colleagues [134] further explained that the EPS component of the biofilm has the tendency to inhibit the activity of antibiotics which disperse through the biofilm, consequently rendering them chelated and forming complexes, or exposed to enzymatic degradation actions, leading to their destruction. This whole pathway is termed diffusion-reaction inhibition [135]. Seemingly, Sharma and coauthors [20] emphasised the vital functions of the component EPS found in the biofilm, which either slows the drug penetration process or reacts with the antibiotics and negatively affects the effectiveness of the drug. Moreover, resistance is observed in the deeper levels of the biofilm where the microenvironment appears challenging owing to the accumulation of metabolic by-products, waste, and nutrients, as well as a reduced level of oxygen, creating an anaerobic environment. A combination of these environmental factors can present with varying effects on antibiotics influenced by the structure and action of the antibiotics. These actions further induce resistance to antibiotics. Seemingly, a high rate of metabolic activity, growth, and protein synthesis occurs at the surface of the biofilm as opposed to no or little rates in the centre, causing minimal penetration and utilisation of drugs in the biofilm.

The multidrug-resistant strains can be transferred to humans through the consumption of contaminated food, which may lead to their spread in the environment. Therefore, monitoring or surveillance of multidrug-resistant *L. monocytogenes* in the food, clinical, and environmental samples could help to identify trends in the prevalence and patterns of resistance to antibiotics, added to enabling the planning and evaluation of approaches to avoid its spread [136]. More elaborately, data on surveillance of antimicrobial resistance indicate the extent/level of current trends in the antibiotic resistance pattern, added to evaluating the effectiveness of the control measures introduced to curb the crisis [4]. Continuous surveillance studies on resistance in this bacterium are necessary due to mutations in bacterial DNA.

##### Persistence of *L. monocytogenes* in the Environment (Food Industry) and Its Implication

Surveys revealed a relatively high occurrence of *L. monocytogenes* in food and food-processing environments in Africa and other countries. The reasons for such levels are ascribed to water quality or inadequate hygiene management in countries [137]. The major fashion of the storage of food in food-processing companies is at refrigeration temperatures, making it unfavourable for the growth of bacteria. However, *L. monocytogenes* survives at these temperatures in these sites, permitting it to grow with minimal competition from other bacteria and thereby become persistent. The persistence of the bacterium in the food-processing facility is described as the strain of this bacterium that survives over time, even following the application of cleaning and sanitation activities. Takeuchi-Storm and colleagues [138] unravelled the persistence of specific sequence types (STs) as they noted the prevalence of *L. monocytogenes* isolates belonging to the same sequence type, exhibiting less than 10 single nucleotide polymorphisms from the same company regardless of the year of sampling and whether the samples were recovered from the environment or products. Generally, enhanced biofilm formation can contribute to the persistence of microbial species in the food-processing environment in the following ways:

1. The microbial species employs biofilm production as a survival strategy. The biofilm-associated bacterial cells are difficult to remove via mechanical means off/from surfaces, and they demonstrate a noticeable reduction in their sensitivity to chemical disinfectants, thus allowing them to resist traditional cleaning methods [139].
2. Owing to the formation of biofilms, bacterial cells can adapt to other environmental stress conditions that usually occur in the food-processing environment such as high salinity and temperature, acidic pH, and UV light [47].
3. Biofilm cells might be more equipped to sequester toxins, cooperate metabolically, exchange nutrients, as well as become more capable of obtaining novel genetic traits, e.g., antibiotic resistance genes, facilitating the survival of the organism in the said milieu [139].

Unrath and colleagues [140] explained that persistence relies on the recurrent isolation of a *Listeria* strain from a facility on different dates over a specified period, which are identified and grouped under the same molecular subtypes. The persistence of this bacterium can be observed from months to multiple years in the food industry owing to the challenges encountered during its control, once persistence is established [132]. This aspect may eventually cause a devastating listeriosis outbreaks as well as a significant cost to food businesses and economies, leading to costly recalls, withdrawals, closures, and loss of reputation [139]. The existence of the bacterium in the environment of facilities that process food is often associated with biofilms. More elaborately, the bacterium’s potential to produce biofilms and a range of strategies to survive and persist in the food environment, occurring on the floors, drainages, equipment, etc., is a cause of concern to industries dealing in food [48,141]. The capability of *L. monocytogenes* to survive in different milieus/habitats is achieved through its adaptation to the different stress conditions (low pH, osmolarity, refrigeration temperature, etc.). The pathogen harbours the two component systems (TCSs) described as sensor and signal transduction modules, comprising a transmembrane sensor histidine kinase (HK) and a cognate cytoplasmic response regulator to sense stresses [142].

The persistence of *L. monocytogenes* in the environment of a facility that processes food is facilitated by its formation of biofilm, posing a significant threat to the safety of food as well as the industry [31]. Lee and colleagues [39] highlighted that the food-processing environment is the most likely means of contamination of different foods with *L. monocytogenes* and the produced biofilms are responsible for the repeated bacterial contamination. Zhang et al. [143] explained that antibiotic-resistant persistent cells can reproduce and disperse from the biofilms, forming new biofilms when antibiotics exert ineffective activity against them. This last stage of biofilm formation releasing planktonic cells represents a great likelihood of contamination or a channel as a potential source of contamination [144]. Persister cells are a characteristic of biofilms. They are described as variant forms of regular cells as they are produced owing to environmental stress conditions that occur within the biofilms. Therefore, they present special characteristics alongside exhibiting a stronger biofilm formation on non-living (abiotic) surfaces in food-associated environments [139]. This is apparent, as the persistent cells assume a dormant state, forming a spore-like structure, and do not divide in the presence of the antibiotics, thus expressing resistance to such an extreme condition. Galie and colleagues [31] noted that biofilms can be formed on contact surfaces, with or without food. Clearly, the bacterium has the capacity to attach to food contact surfaces, comprising polystyrene, glass, and stainless steel, persisting even for several years in the food industry, which can eventually cause the recurrent cross-contamination of the food products manufactured in that facility. Considering the diverse strains of the bacterium, *L. monocytogenes* has the ability to form biofilms, and the architectures of the biofilms vary among the strains [144]. The organism’s ability to form biofilms at low temperatures that are usually employed in the processing and storage of foods promotes the probability of cross-contamination. In the food industry, described as a natural environment, the formed biofilms consist mostly of multiple bacterial species, indicating that *L. monocytogenes* form multi-species or mixed-species biofilms which have demonstrated greater resistance to disinfectants andsanitisers. Hence, multi-species biofilms are highly stable and not easily controlled, causing the organism to persist for years in the food facility, thus increasing the risk of contamination of the food-processing facility and ultimately the food, resulting in the spoilage of food which will eventually affect the consumer’s population and cause severe economic concerns [45].

On the other hand, Leong and coauthors [91,109] highlighted the responsibility of harbourage sites in the persistence of *L. monocytogenes*. A harbourage site is described as an area into which sanitation agents do not properly penetrate, or it can be said to be devoid of the disinfectant; therefore, the bacteria are not removed properly. Another suitable definition of a harbourage site is an area where the disinfectant reaches but in a lower concentration and does not get properly dried, causing the substance to occur and remain on the site at a concentration which is sublethal, thus offering ample time to the bacterium to build a resistance against the product and, subsequently, a community of *L. monocytogenes* is developed, exhibiting resistance against the cleaning products. Therefore, from this site, the strain can be disseminated to other parts of the facility [145]. Apparently, several of the antimicrobials applied for biofilm removal in the industry only reduce and inactivate the microorganisms, thereby causing persistence. Accordingly, Carpentier and Cerf [145] associated persistence with failure of removal of cells from niches/milieus within the food environment that are regarded as difficult or hard-to-clean points. Consequently, the organism can survive, grow, and is able to colonise other surfaces producing biofilms, as revealed by several researchers, identifying the bacterium on floors, walls, conveyor belts, bends in pipes, rubber seals, stainless steel, drainages, as well as improperly cleaned and equipment and areas, others [12]. Furthermore, *L. monocytogenes* cells in biofilms are said to observe physiological changes. Colagiorgi and colleagues [144] mentioned that owing to the presence of gradients of nutrients, oxygen, and other molecules, there is bound to be a structural, chemical, and biological heterogeneity in a biofilm, thus explaining that the properties of biofilms are highly variable among the bacterial strains, which in turn exert effects on the risk of contamination of food [109,141]. The risk of contamination can equally be influenced by the transfer of biofilm cells to the food matrices, which is dependent on the properties of the biofilm alongside the factors that may condition them.

Due to the complex ecology of *L. monocytogenes* and its ability to survive in the adverse environmental conditions that occur in the food-processing environment, the bacterium remains a public health concern. Its adaptation, survival, and long-term persistence in the said milieu is attributed to the bacterium’s ability to multiply at a low temperature, pH, and osmotic stress, alongside its displayed resistance to sanitation agents as well as biofilm formation [45]. In addition, *L. monocytogenes* adapts to such environments through its ability to tolerate toxic metals (cadmium and arsenic). These actions of resistance are usually associated with the expression of resistance genes. Therefore, the co-occurrence of resistance genes to both toxic metals and biocides in the bacterium enables the selection of different resistance genotypes and phenotypes that can eventually cause listeriosis in humans [132]. On the other hand, Meesilp and Mesil [146] mentioned that biofilm-forming bacteria, for example, *L. monocytogenes*, can provoke technical challenges in the food industry, leading to a loss of production efficiency because they may inhibit heat flow across the equipment, enhance the frictional resistance at the surfaces, and promote the corrosion rate of the surfaces.

In a nutshell, the persistence of the organism in the food-processing environment could be attributed to numerous factors acting externally, and include insufficient or ineffective sanitisation practices, the tendency to grow at low temperatures, resistance to heavy metals, alongside the possibility of the availability of specific genes responsible for biofilm production in some strains of the bacterium [39,147]. The persistence of *L. monocytogenes* in the food-processing environment has become the major cause of post-processing contamination and cross-contamination owing to the challenge of not completely eradicating the bacterial cells from the food environment. This, in turn, is very pivotal for the transmission of the bacterium to food matrices as well as humans [148]. Therefore, having knowledge and insight into the adaptation techniques displayed by *L. monocytogenes* to stress factors emanating from the environment will lead to the advent of novel, effective, and cost-effective methods for controlling the pathogen in food production facilities. In other words, unravelling the factors/sources that drive the persistence of *L. monocytogenes* in the environment will help in the development of more target-oriented control and prevention approaches [45].

The organism’s persistence in the food-processing facilities through the formation of biofilms can aid the cause of infections in humans. Like microbes, the body’s system recognises the biofilm as foreign and, immediately, it triggers an immune response via immune cells, receptors, and several humoral factors [149]. Rather and colleagues [16] noted that microbial biofilms escape the body’s immune system, a situation leading to long-term persistence. The formation of biofilm provokes the development of antibiotic resistance and the formation of persister cells, which makes the persistence of infections caused by microbes uncontrollable [150]. This is explained by Donelli and coauthors [151], who highlighted that biofilms present huge pathological manifestations and are found to be everywhere, inhabiting both biotic and abiotic surfaces (e.g., medical implants, living tissues, water channels, pipes, hospital floors, food-processing units, among others). On the other hand, Sharma and colleagues [20] stated that biofilms can cause infections in the following ways: (a) the interchange of resistance plasmids between cells that occur within the biofilm, (b) reduced sensitivity to antimicrobial agents by the cells, (c) the production of endotoxin by the biofilm-associated bacteria, d) detachment of cells or masses of cells of biofilm into the food, or unto other surfaces and equipment, or even humans working in the processing facility, and (e) resistance to the immune system of the host.

#### 2.1.2. Control of Biofilms in Food Industries

Worries pertaining to contamination with *L. monocytogenes* are on the rise following the increased consumption of ready-to-eat foods, for example, meat products. Meats are a milieu rich in nutrients that facilitate pathogen growth and reproduction [21]. Biofilm formation is observed as one of several ways via which the organism exhibits resistance to antibacterial agents and disinfectants along with persisting in the food chain [39]. Of the greatest advantage of the biofilm environment is the closeness of multiple species of bacteria to one another, making it easy for them to communicate through quorum sensing and promoting the dissemination of mobile genetic elements. Most of the transposable DNA are associated with biofilm-producing potential, and the matured biofilms disperse planktonic *L. monocytogenes* cells into the environment to begin a new cycle of biofilm formation, therefore, causing the sustainability of biofilms and, consequently, contributing to the spread of bacterial contamination in the environments of facilities that process food [152]. Biofilms in the said environment are considered as avenues for cross-contamination of foodstuffs. Thus, biofilms are said to be responsible for most of the outbreaks of food-borne diseases and food poisoning. Owing to the protection rendered by the self-produced matrix to the microbial cells within an established biofilm, it is quite challenging to eradicate the bacterial cells [39].

In this light, control strategies are eminent in order to provide effective solutions to eliminate the established biofilms and to control/prevent their formation, especially as *L. monocytogenes* has the tendency to form these structures and persist in food facilities [48]. According to Mazaheri and coauthors [48], the strategies to control biofilm formation vary across different food industries, ranging from fish, dairy, chilled foods, vegetables, and meat to ready-to-eat products. Therefore, the best practices for addressing contamination with *L. monocytogenes* in a food manufacturing facility are through implementing a sanitation schedule for the facility, creating an environmental monitoring programme, as well as testing the finished products [153]. According to Holah et al. [154], sanitation offers the following to the food-processing industries: it helps to remove visible soils and allergens, alongside removing microorganisms that may alter the organoleptic characteristics of the food. Cleaning and disinfection comprise the two major aspects of the hazard analysis and critical control points system [48]. The authors further emphasised that cleaning and disinfection of industrial surfaces helps in controlling and preventing cross-contamination of meat by *L. monocytogenes*.

Recently, the application of lysozyme, a naturally occurring enzyme in egg whites, as a control strategy against biofilms has attracted great interest. The enzyme is known to hydrolyse the β-1,4 glycosidic bonds of the peptidoglycan layer in the cell wall of Gram-positive bacteria, thus inhibiting their growth [155]. Lysozyme is highly safe and exhibits stability over broad pH and temperature ranges, demonstrating a great prospect in food processing and preservation [156]. In the same vein, Nguyen and Burrows [157] mentioned that the biofilm produced by *L. monocytogenes* consists mainly of proteins; therefore, the application of protease treatments triggered a variation in the development of biofilms. Shen et al. [158] reported the importance of food additives in the prevention of biofilm formation. They explained that monascus pigment (a red intracellular pigment produced by the fungi, *Monascus* spp., via fermentation) and polylysine (homopolymer) possess a wide antibacterial level, for which the substances are largely implemented in food processing and preservation to thwart microbial growth by altering the interactions between the bacterial cells and surfaces. This alteration inhibits the formation of biofilms by the bacterial cells [158].

Apparently, monitoring bacterial contamination either in planktonic forms or as biofilms in foods is paramount. Even though biofilms containing *L. monocytogenes* have been emphasised as the specific hazardous source of contamination to RTE food, the planktonic state appears as the current model for decision-making [159], more especially because any strain is said to have the capacity to produce biofilms on any surface with any environmental conditions, despite varying biofilm properties occurring between the different strains [141]. Consequently, the lack of thermal treatment of RTE foods prior to consumption presents these foods at a high risk of contamination. Mazaheri et al. [48] highlighted the use of natural organisms or controlled microorganisms or their antimicrobial products to extend the shelf life or enhance the microbiological safety of foods. This is referred to as biocontrol, wherein two biological agents are employed—that is, bacteriophages (viruses that infect bacteria) and lactic acid-producing bacteria (LAB). Specifically, *Listeria* phage P100 was employed by Gray and colleagues [160] to eliminate biofilms that occurred in processed meat products and on food contact surfaces in processing industries. Furthermore, Webb and colleagues [161] reported the use of lactic acid bacteria (LAB) in the biological control of *L. monocytogenes* in RTE foods. The authors further highlighted that LAB occur naturally as microflora in RTE foods, employed to extend the shelf life of the products as well as to prevent pathogen proliferation through growth competition and the production of metabolites.

### 2.2. Acid Tolerance

*L. monocytogenes* thrives in distinct food matrices, where they become subjected to different acid levels. Moreover, acidification of food (including dairy, meat, and vegetables) is one of the methods of preservation in the food industry, which occurs primarily through fermentation by bacteria present in the food or bacteria added as starter cultures [162]. In addition, there is the widespread use of organic acids in the food industry to control the growth of microbial pathogens, as it is explained that the accumulation of acid anions intracellularly is the key inhibitor of cell viability [163]. Mani-Lόpez et al. [164] recorded that the internal accumulation of acid anion is the major process through which the organic acids demonstrate their antimicrobial activity. This practice results in acidic conditions, which is tantamount to increased hydrogen proton concentration, resulting in the growth inhibition of bacterial cells. Moreover, following the ingestion of food contaminated with the bacterium, the organism travels through to the stomach, where it becomes subjected to the acidity of this site.

One of the physicochemical factors influencing the behaviour of microorganisms in every environment or habitat or niche is acidity, and the response of these microbial species to acidity usually affects their ability to grow and survive [165]. The authors further mentioned that unicellular organisms, including bacterial cells, are often fraught with the specific challenge of a high concentration of protons (acidic conditions) because the protonation of biological molecules can have impacts on their structure, charge, and function, resulting in serious damaging effects on the cells. Acid stress responses can be grouped into acid tolerance that describes the persistence and growth of the organism at moderately low (pH 4–6) pH and acid resistance that describes the survival at extremely low pH (pH 2–4) [166], consisting of a complex phenotype [165]. Notwithstanding this, *L. monocytogenes* is said to adapt to the acidic conditions in the environment via the use of various metabolic and homeostatic mechanisms to ensure that the intracellular pH is maintained within a certain range that is ideal for its growth and survival [165].

In detail, the organism utilises the glutamate decarboxylase system (GAD encoded by *gadD1*, *gadD2*, *gadD3*, *gadT1*, and *gadT2*), the arginine deiminase (ADI) pathway, plus the function of another proton pump (F_0_F_1_-ATPase) to increase the buffer capacity of the cytoplasm [167,168]. According to Smith et al. [169], GAD and ADI are two enzymatic systems engaged in the regulation of the concentration of internal hydrogen ions. Nevertheless, Wiktorczyk-Kapischke et al. [61] highlighted that the adaptation systems of *L. monocytogenes* (including acid tolerance response, (ATR) GAD, ADI, and F_0_F_1_-ATPase) operate at the same time, ensuring that the bacterium survives and adapts to the acid stress conditions. These allow the bacterium to thrive in such conditions, alongside shielding the organism from other adverse conditions found in the medium/milieu and increasing its virulence [170]. So far, the alternative sigma factor sigma B, the RofA-like transcriptional regulator GadR, and the arginine repressor ArgR are the three major regulators described in the transcriptional reprogramming needed for the metabolic changes involved in the acid response process [166].

In addition, adaptive acid tolerance response (ATR) is one of the mechanisms employed by *L. monocytogenes* to survive in acidic conditions [171]. Koutsoumanis et al. [172] purported that the growth of *L. monocytogenes* in a moderate pH medium could heighten its resistance to the lethal pH of the stomach; this occurrence is termed acid tolerance response. It is the persistence or growth of the bacterium at moderate acidic conditions between 4 and 6. In detail, a brief adaptation period at a non-lethal pH is observed, which induces changes in the metabolism of cells, allowing the organism to survive in acidic conditions. More elaborately, Liu and colleagues [57] reported the induction of ATR in three strains of *L. monocytogenes* with the use of lactic acid and that the inducible acid resistance observed a decrease with an increasing extracellular pH, but with the strongest ATR in cells induced at an extracellular pH of 5.5.

Clearly, the GAD system consisting of five genes arranged into three separate genetic loci (*gadDITI*, *gadT2D2* and *gadD3*) is recognised the primary mechanisms utilised by *L. monocytogenes* to maintain its intracellular homeostasis, wherein *gadD1*, *gadD2*, and *gadD3* genes encode decarboxylase, meanwhile *gadT1* and *gadT2* are implicated in antiporters production. The glutamate decarboxylase enzyme operates while encouraging the conversion of cytosolic glutamate to a neutral compound known as gamma (γ) aminobutyrate (GABA) in an irreversible fashion. While producing GABA, the proton level experiences a decrease intracellularly, leading to a corresponding alkalisation of the milieu or medium, as well as a rise in the internal pH of the *L. monocytogenes* [45]. Considering a variation in the acid level/acid conditions and severe acid conditions, the glutamate decarboxylase GaD1/GadT1 systems become operational and GaD1/GaDT1 is significant for the organism’s survival, respectively [173]. In addition, the ADI system consists of arginine deiminase, (ArcA) which functions in tandem with two other enzymes (catabolic carbamoyl transferase, ArcB, and carbamate kinase, ArcC) and the arginine/ornithine antiporter (ArcD), converting external arginine to ornithine. This reaction occurs in two steps, releasing other substances such as carbamoyl-phosphate and ammonia. Seemingly, carbamoyl-phosphate is further converted to ammonium and carbon dioxide with the release of ATP via the activity of carbamate kinase, ArcC [165].

The compound/substance ornithine is transported extracellularly via a membrane-bound antiporter in an energy-independent way, in exchange for an arginine molecule. The system releases ammonia as a by-product to a level which combines with intracellular protons, yielding ammonium ion (NH4+). This reaction helps to maintain the cytoplasmic pH at the intracellular level, in that way sheltering the bacterial cells from the harsh acidic extracellular milieus [174]. Subsequently, ATP produced during the ADI pathway is employed in the F_0_F_1_-ATpase (proton extrusion) pathway. F_0_F_1_-ATpase is an enzyme harbouring two distinct domains; F_0_ denotes a channel for proton translocation, and it is the membrane domain, whereas F_1_ is the cytoplasmic domain involved in increasing the rate of synthesis and hydrolysis of ATP molecules [169]. Apparently, ATP produced following the conversion of arginine through the ADI system is employed by the F0F1-ATPase to produce a proton gradient that favours the expulsion of hydrogen ion (H+) and the restoration of homeostasis [169]. The authors further highlighted that *L. monocytogenes* with the help of the above-mentioned adaptation mechanisms, can combat the challenges presented by acidic conditions, including the gastrointestinal tract and acidic foods. Therefore, Saklani-Jusforgues and colleagues [169], in their findings, suggested that acid adaptation of the bacterium in acidic (low pH) foods, alongside keeping the gastric pH level under control via dietary practices or the application of inhibitors of gastric acid secretion, may be a potential risk factor aggravating the onset of listeriosis in humans.

In an acid stress condition, the bacterial cell can equally decrease the destructive effects of the acidic conditions on membranes, proteins, and DNA by deploying various protective and repair processes [165]. The bacterial cell of *L. monocytogenes* is endowed with an atypically high level of branched-chain fatty acids (BCFAs). The organism’s capacity to modulate the relative fractions of the various BCFAs permits its adaptation to moderate pH stress [175]. Dps is a DNA-binding protein that is widely distributed throughout the domain, bacteria [176]. Its homologue Fri exists in *L. monocytogenes*, and it is viewed as the principal cold shock protein, participating in the organism’s virulence, adding to the role it plays against multiple stresses [177].

### 2.3. Thermotolerance

*L. monocytogenes* can grow and survive in a vast temperature range of −0.4 to 45 °C, indicating that the bacterium can tolerate both low and high temperatures in the environment where it is found [178,179]. The organism has been isolated from refrigerated foods severally, explaining its capacity to thrive at low temperatures offered under refrigeration conditions during storage. Therefore, the bacterium is termed a psychrotroph with the tendency to withstand refrigeration at −1.5 °C. Roberts et al. [179] registered that refrigeration is a conventional technique often employed to extend the shelf life of certain foods, which greatly alters the physiological and biochemical mechanisms of the pathogenic bacteria in response to the low-temperature conditions [180], thus emphasising the necessity to comprehend how *L. monocytogenes* can survive these conditions. Several researchers described the phenomenon of this mechanism in response to low temperatures to be complex, involving the uptake of cryoprotective compounds from the environment, the expression of cold shock proteins, and alterations in the composition of the cell membrane, added to a reduction in bacterial cell metabolism [181]. Therefore, it is apparent that *L. monocytogenes* responds to low temperature conditions in varying ways, which causes the bacterium to modulate its gene expressions, particularly those responsible for cell membrane function, lipids, carbohydrates, and amino acid synthesis, alongside the genes implicated in movement and biogenesis [182].

Once the organism is subjected to a low temperature, this condition exerts an effect on the cell, resulting in a decrease in the lipid fluidity of the membrane [183]. As a response, *L. monocytogenes* alters the lipid composition of the membrane, increasing the concentration of unsaturated fatty acids, which helps to prevent the formation of a gel-like state that may lead to the leakage of the content of the cytoplasm [45]. NicAogaín and O’Bryne [184] explained that the organism creates the optimum membrane fluidity for the activity of enzymes and transport through the membrane. In addition, the authors noted that the rate of enzyme activity in the intracellular space is decreased to the required minimum and the fitness of the cell is improved. In the same vein, Angelidis and Smith [185] reported that the bacterial cell increases the accumulation of organic osmolytes (glycine betaine and carnitine) from the medium/milieu/environment by the chill-activated transport system. Furthermore, Hébraud and Guzzo [186] mentioned the expression of cold-shock domain family proteins (these are structurally related proteins of 60–70 amino acids in length) following *L. monocytogenes*’ exposure to low temperatures. These cold shock proteins (Csps) are said to be extensively distributed among prokaryotes [187], stabilizing the conformation of nucleic acids, as well as acting as molecular chaperons and facilitating vital processes taking place in the bacterial cell such as replication, transcription, and translation at low temperatures [181]. Specifically, Muchaaamba and colleagues [188] demonstrated the contribution of CspA (among the numerous Csps that have been identified thus far) to the resistance of this organism against low temperatures.

Overall, RTE foods are of high concern at this juncture because they are always stored at low temperatures, which permits the growth of *L. monocytogenes*, and they are not subjected to any subsequent treatment that could inactivate it prior to consumption [183]. The authors further expressed that under the low-temperature conditions (e.g., 5 °C) and in the presence of exogenous unsaturated fatty acids, there appeared to be a high level of incorporation of unsaturated fatty acids, decreasing the weighted-average melting temperature of the fatty acids found in the membrane, thus permitting *L. monocytogenes* to counterbalance the decrease in fluidity caused by temperature, hence resulting in increased growth. Thermal treatment is among the procedures employed to hinder or reduce bacterial growth in food production and preservation. This equally applies to *L. monocytogenes*. However, the natural resistance of *L. monocytogenes* to increased temperatures exceeding 45 °C was noted, which was said to negatively influence the efficacy of thermal inactivation of the bacterium during food processing [189].

The resistive tendency of *L. monocytogenes* to heat is affected by certain external factors, including the age of the bacterial cell, strain serotype, the composition of the food, along with previous growth and stress conditions [190]. Following *L. monocytogenes*’ exposure to raised temperature, the bacterium demonstrates a rising production of heat shock proteins (HSPs) that are encoded by three different classes of heat-shock-associated genes, including class I, class II, and class III that exhibit different functions [61]. Class I HSps are encoded by several genes, for example *grpE*, *dnaK*, *groEL*, and *groES*—serving as intracellular chaperons and functioning to stabilise and repair proteins that are partly denatured, alongside avoiding their intracellular aggregation under heat stress conditions [181]. Meanwhile, the class II HSp genes encode class II HSps, which are general stress proteins that are transcribed, depending on the alternative sigma factor SigB in varying growth-inhibiting conditions [191]. Also, class III HSps are encoded by class III HSp genes, including *clpP*, *clpE*, and *clpC* operons being negatively regulated by the class III HSP gene regulators. The resultant class III HSps, ClpC, and ClpE, are said to belong to the heatshock protein Clp family of highly conserved molecular chaperons, possessing ATPase activity, while ClpP is a serine protease protein exhibiting proteolytic activity [192]. The tolerance of *L. monocytogenes* to heat is said to be strain specific. Lakicevic et al. [59] affirmed that the high level of strain divergence is said to influence virulence potential, adaptation to the environment, and responses to stress factors. Accordingly, Doyle and colleagues [190] reported that exposure to other stress factors before heat treatment could permit *L. monocytogenes*’ tolerance to heat.

### 2.4. Osmotic Shock

Salt is among the agents employed in the preservation of some foods, e.g., meat with the reasoning that it increases osmolarity, leading to the suppression of bacterial growth. Amezaga et al. [193] explained that bacterial cell inhibition is ascribed to decreasing water activity in the surrounding milieu, facilitating plasmolysis, which leads to decreased intracellular turgor pressure as an effect. *L. monocytogenes* demonstrated growth at a salt concentration of up to 10%. Therefore, it can experience osmotic stress at different temperatures, while its adaptation to osmotic stress or response to osmotic stress is said to be in a temperature-dependent manner [194]. The response of *L. monocytogenes* to osmotic shock is described as osmoadaptation. This process involves both primary and secondary response mechanisms, thus it is termed a biphasic process [195]. In scenarios of elevated osmolarity, the primary adaptation involves bacterial cells undergoing physiological changes, maintaining their turgor through elevating their uptake of potassium ion (K+) alongside glutamate (counterion) into the cell. Two K^+^ transporters, described as a high-affinity KdpABC transporter system and a low-affinity system, are said to play a major role in response to a high salt level [196].

In the secondary response or the second phase of osmoadaptation, the bacterial cell replaces part of the accumulated K+ with low-molecular-weight molecules that are described as osmolytes or compatible solutes [197]. The author further explained that this action or the uptake of the osmolytes aids in restoring the turgor pressure and cell volume in *L. monocytogenes* as well as stabilising the structure and functions of cell proteins. A host of compatible solutes were identified, viz. betaine, carnitine, proline, gamma butyrobetaine, etc., that are responsible for encouraging tolerance to both salt and low temperatures [198].

The subjection of *L. monocytogenes* to osmotic stress conditions may result in cross-protection against other stressors [194]. This is obvious when the bacterium demonstrates resistance to osmotic shock via releasing compatible solute transporters and cold shock proteins, substances that equally contribute to the resistance of the bacterium to low temperatures, thereby causing it to grow [194]. Wiktorczyk-Kapischke and colleagues [61] investigated the effect of certain stress factors on the growth and survival of *L. monocytogenes*. The authors registered that those five tested strains of *L. monocytogenes* survived exposure to a temperature of 70 °C for 60 min, 62% of the strains were inhibited appreciably with 8% sodium chloride salt (Nacl), and all the strains remained viable at every phase of the cold stress experiment. Conclusively, the information assembled on the survival of *L. monocytogenes* in stress conditions forms the baseline for one of the hypotheses that clarifies the development of persistent strains. Bergholz et al. [194] explained that the organism’s potential to grow and proliferate under the defined stress conditions contributes to the origin of the persistence of *L. monocytogenes* in foods and food-processing facilities, therefore heightening its transmission to humans through food. This is because of the challenges encountered during the control throughout the entire food chain from production to storage and consumption.

Therefore, a thorough understanding of the different mechanisms or processes utilised by the organism to tolerate these stress conditions in the food matrices and food-processing facilities is paramount for the efficient development of measures that would prevent contamination during processing and control growth during storage of the food [184].

## 3. Virulence Factors, Strain Variation, and Pathogenesis of *L. monocytogenes*

*L. monocytogenes* is a major zoonotic, food-borne pathogen, endowed with a myriad of virulence traits that are either scattered across the genome or clustered in pathogenicity islands (LIPI-1, LIPI-3, LIPI-4) and functioning in the pathogenicity of the organism (Table 2). An assortment of virulence factors known as proteins are found in the bacterium, consisting of internalins (inl A and inlB), fibronectin-binding proteins (Fbps), *Listeria* adhesion proteins (LAPs), phospolipases, listeriolysin (LLO), and actin polymerising protein (ActA) [199]. In addition, inlA and inlB are both recognised as major adhesion proteins encoded by the *inlA-inlB* locus that are found attached to the cell wall via the C-terminal LPXTG motif, as well as through glycine and tryptophan, respectively. These proteins are encoded by virulence genes, including *prfA*, *hyl*, *Act*A, *plc*A, *plc*B, and *mpl*, found on the *Listeria* pathogenicity island (LIPI-1) conserved in the genomes of all strains of *L. monocytogenes* [200]. Coincidently, LIPI-1 and the *inlA-inlB* locus are very crucial to the pathogenicity of the organism, being essential for the major steps of intracellular parasitism, thus explaining their conservation in almost all strains of the pathogen [142].

According to Quereda et al. [142], *L. monocytogenes* exhibits an unparalleled ability to detect and react to environmental stresses/stimuli, permitting it to be transmitted from contaminated foods into the gastrointestinal tracts of human hosts. The bacterium causes a food-borne infection in humans, termed listeriosis [201]. The likelihood of acquisition of the infection varies and is influenced by the exposed subpopulation and the strain of the *L. monocytogenes* ingested [202]. Contrary to other food-borne diseases, listeriosis is of low incidence, occurring the vulnerable/susceptible population, constituting newborns, pregnant women, immunocompromised individuals (HIV/AIDS patients), the elderly, and people with underlying diseases (kidney disease, cancer, and diabetes). *L. monocytogenes*-contaminated, ready-to-eat foods are responsible for the infection [202]. Following the ingestion of these foods, the gastrointestinal tract becomes colonised by *L. monocytogenes* and the bacterium travels through the intestine and enters the body, creating the possibility of spreading through the blood to other target organs, including the liver, central nervous system, spleen, and the foetus [142]. The authors remarked about the versatile nature of the pathogen, displayed by its exceptional approach to invade diverse cell types, capacity to survive and exhibit motility within eukaryotic cells of the host, as well as its being disseminated from cell to cell. This describes the organism’s capacity to travel through three significant barriers in the human host, including the intestinal epithelium, blood–brain barrier, and placenta, with subsequent dissemination to other organs [203].

More elaborately, *L. monocytogenes* possesses the capacity to promote its self-internalisation through host cells, resulting in the successful induction of the infection [204]. Adhesion and invasion of host cells, internalisation by host cells, lysis of the vacuole, intracellular multiplication, and intercellular spread to neighbouring cells are the different stages at which the different virulence factors perform their functions. These stages represent the infection process of the host with *L. monocytogenes* [205]. Occurring as an intracellular pathogen, it relies on a repertoire of adhesion and invasion factors to enable its colonisation of the gastrointestinal tract as well as its transit via the intestinal barrier. Once introduced into the host system via ingestion of contaminated food, the pathogen becomes subjected to high acidity, bile salts, non-specific inflammatory responses, and proteolytic enzymes; however, it survives the generated responses. Inside the intestines, a bacteriocin encoded by a pathogenicity island, LIPI-3, is highly expressed, provoking the alteration of the microbiota in the host’s intestine, which results in the colonisation of the intestine by the bacterium [206]. The stress-induced adaptive tolerance responses to acid and osmotic and oxidative stresses that take place in the food environment are like those occurring in the GIT, thus protecting the pathogen against such stresses and directly impacting on its pathogenicity potential [207]. Accordingly, Sibanda and Buys [199] mentioned that the reprogramming of genes that are expressed in response to stress, to enable survival of *L. monocytogenes* while in the GIT, to virulence-related genes, permits the bacterium to switch from an avirulent to virulent state. In addition, the authors further affirmed the overlapping function and indirect and direct interactions between the general stress response regulator, the Sigma factor B (SigB), and the positive regulatory factor A (prfA) that controls the transition or switch.

Following its survival, the organism becomes attached and enters into both the phagocytic and non-phagocytic cells, aided by surface proteins known as internalins [204]. Although phagocytic cells are endowed with the capacity to destroy ingested bacteria recognised as foreign to the body, *L. monocytogenes* can survive within these cells, thus contributing to its pathogenicity. Following the organism’s adherence to the epithelial tissue of the gastrointestinal tract, *L. monocytogenes* becomes internalised by the macrophages in the phagosomal vacuole, from where it escapes with the help of listeriolysin O (LLO, a cytolysin) and phosphatidylinositol-specific phospholipase (plcA). While in the host, the availability of sufficient nutrients causes the organism to replicate in the cytoplasm and the organism is moved intracellularly across the cytoplasm to adjacent cells, thus disseminating the infection with no exposure for the second time to the extracellular immune surveillance of the host [208]. This movement is facilitated by an actin-based motility machinery (e.g., actin polymerisation protein, ActA, a bacterial surface protein) found in the host cell. In the neighbouring cells, *L. monocytogenes* becomes internalised and enclosed by a double-membrane vacuole; however, being aided by LLO and phoshatidylinositol-specific phospholipase (plcB), the organism is set to start its life cycle again [201].

**Table 2:** Description of the virulence factors in *L. monocytogenes* and their roles performed during pathogenesis.

Virulence Factors	Descriptions	Genes Responsible	Functions	References
Internalins InlA	This is a major virulence factor of the bacterium of molecular weight (80-KDa), attached to the cell wall.	*InlA-InB* locus	It mediates the uptake of *L. monocytogenes* into non-phagocytic cells and promotes the adhesion and invasion of the intestinal epithelium by interacting with E-cadherin receptors.	Dellafiora et al. [209]; Ireton et al. [210].
InlB	Together with inlA, inlB is equally a major adhesion protein that is attached to the cell wall and interacts via non-covalent bonds with the teichoic acid component of the cell wall.	*InlB-InlA* locus	The protein helps in the adherence of the pathogen and invasion of the intestinal barrier.	Ireton et al. [210].
*Listeria* adhesion protein (LAP)	A cell wall protein (104-KDa) described as an alcohol acetaldehyde dehydrogenase that is produced predominantly as a cytosolic protein. It is an essential enzyme that occurs ubiquitously in all *Listeria* species.	Not applicable	In pathogenic *Listeria* species, LAP is translocated to the surface of the cell via the SecA2 secretory system to enable the adhesion of the pathogenic species to the intestinal cells. It equally helps in the translocation of the pathogenic cell across the intestinal epithelium.	Burkholder et al. [211]; Drolia et al. [212].
Fibronectin-binding protein (FbpA)	This is a 570-amino acid polypeptide that is attached to the cell wall but exposed on the cell surface with no signal peptide. It is similar in homology to Fbp in *Streptococcus*. The Fbps are widely distributed in Gram-positive bacteria.	Not applicable	They recognise and bind to fibronectin, forming a three-component bridge, enabling the adhesion of the bacterial cells to the host cells.	Henderson et al. [213]; Hymes et al. [214].
Actin polymerisation protein (ActA)	This is a surface protein that is attached through its hydrophobic C-terminal domain to the cell membrane of the bacterium, whereas its N-terminal domain is exposed to the cytoplasm host cell.	*ActA*	The protein demonstrates an asymmetrical distribution, influencing the directionality of the bacterium’s motility. Secondly, it recruits a host of vasodilator-stimulated phosphoprotein (VASP) and actin-related proteins-2 and 3 (Arp2/3) complex to facilitate filament formation and actin nucleation.	Suárez et al. [215]; Kühn and Enninga [216].
Listeriolysis (LLO)	LLO is a 56-kDa pore-forming cytotoxin, belonging to the cholesterol-dependent cytolysin (CDC) family.	*Hly* gene	Responsible in the lysis of internalisation vacuole that leads to the discharge of the pathogen into the cytosol of the host cells.	Hamon et al. [217]; Phelps et al. [218].
Phospholipases (two types) Phosphatidylinositol-specific phospholipase C (PI-PLC) Phosphatidylcholine phospholipase C (PC-PLC)	PI-PLC	*PlcA* gene	PI-PLC complements LLO in the lysis of the primary and secondary vacuole after the internalisation of the pathogen. It provokes the splitting of the phosphatidylinositol membrane into inositol phosphate and diacylglycerol.	Pizarro-Cerdá et al. [219].
	PC-PLC is a 29-KDa enzyme, broad ranged in nature, and it is formed from a precursor of 33-KDa through cleavage but requiring a zinc-dependent metalloprotease for maturation.	*PlcB* gene	In an LLO-deficient milieu, PC-PLC is involved in the lysis of the double-membrane secondary vacuole and the primary vacuole.	Coffey et al. [220]; Gründling et al. [221].
Positive regulatory factor (PrfA)	PrfA is formed from three promoters, including Sigma B (SigB).	LIPI-1	It induces the transcription of LIPI-1, the predominant virulence regulon, acting as the major regulator of virulence factors that enable intracellular replication and bacterial spread to neighbouring cells.	Quereda et al. [142]; Tiensuu et al. [222].

**Figure 2:**
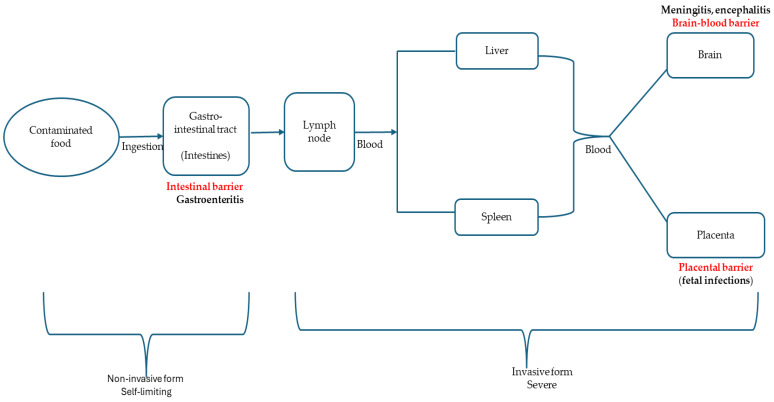
Successive steps in the clinical manifestations of listeriosis showing the 3 significant barriers in a human host.

Listeriosis can manifest in two forms, including the non-invasive febrile gastroenteritis (self-limiting) and severe invasive listeriosis that is associated with more severe symptoms, comprising encephalitis, meningitis, endocarditis, sepsis, pneumonia, septicaemia, meningoencephalitis, and brain infection [223]. Spontaneous abortions or stillbirths can equally manifest due to cervical or intrauterine infections caused by *L. monocytogenes*. Figure 2 displays the clinical manifestation of listeriosis, focusing on the different forms of listeriosis added to the three significant barriers in human hosts that lead to the severe and invasive listeriosis. Clinical manifestations of both forms of listeriosis are based on the age of the person, the immune status of the individual, the virulence of the ingested *L. monocytogenes* strain and physiological state, plus the infectious dose and mode of infection [224]. Accordingly, the non-invasive febrile gastroenteritis affects the immunocompetent population, causing atypical meningitis and septicaemia, and it is associated with headache and backache, alongside fever and watery stool that lasts for 2–3 days [225]. However, the symptoms are self-limiting, and the patient can recover within a fleeting time, without seeking diagnosis by a clinician; even when diagnosis is sought, the non-invasive listeriosis is missed owing to the less severe manifestations. This leads to under-reporting of listeriosis cases in developing countries [226].

### Strain Variation/Diversity in L. monocytogenes

Genotyping studies performed on a large scale among clinical, food, and environmental isolates provided great knowledge about the structure and distribution of the *L. monocytogenes* population. Epidemiological studies suggest that the bacterial species is highly pathogenic, and it is said to be heterogenous relating to virulence. The opportunistic pathogen *L. monocytogenes* can be categorised into four divergent lineages: I, II, III, and IV, through the multilocus sequence typing of internal portions of seven housekeeping genes. Lineages I and II are classified as the major lineages, while lineages III and IV are described as minor lineages [227]. Considering the distribution of genotypes among the clinical isolates, Koopsman and colleagues [228] noted an unequal distribution with 2/3 belonging to lineage I and 1/3 associated with lineage II. Belonging to the different lineages are specific serotypes grouped based on agglutination of somatic (O) and flagella (H) antigens. In total, 14 serotypes exist and 4 h is the most recently identified. Further characterisation and differentiation results in genetically highly similar stains termed clonal complexes [229]. The clonal complexes can be differentiated into hypervirulent and hypovirulent clones with differing adaptability/abilities; the hypervirulent clones demonstrate a better ability at colonising the intestine and exhibit a higher invasion rate of the intestinal mucosa, unlike their other counterparts [142]. Meanwhile, hypovirulent clones are well-adapted to the food-processing environments and express higher degrees of resistance and tolerance genes to stressful conditions and disinfectants, respectively [230]. The genetic evolution of the bacterium is as presented in Table 3.

The heterogeneity in *L. monocytogenes* was suggested through epidemiological and experimental studies to be responsible for the variability in environmental distribution, virulence, as well as clinical manifestations observed between the different hosts [227].

The virulence of the bacterial strain tends to affect the severity of listeriosis manifesting in humans, alongside other factors, including the infectious dose, the overall degree of health and immunity of the infected host, the age of the infected person, the diversity of the genetic background of the population, and any characteristics of the food that cause changes in the status of the microbes or host [45]. In addition, Zelalem and Dima [231] reported that the time of food storage, temperature, product type, and the traditional consumption of raw foods are among the risk factors of infection. Only three serotypes (1/2a, 1/2b, and 4b) are known to cause more than 95% of listeriosis in humans [232]. Table 4 below shows the different disease outcomes or clinical manifestations, indicating the severity of the listeriosis as well as the speculated infectious dose, as the case may be.

From Table 3 and Table 4, it can be recommended that the individuals categorised as high-risk groups, collectively termed YOPI (e.g., young, old/elderly, pregnant women, immunocompromised, etc.) should avoid consuming food with a great likelihood of high concentrations of *L. monocytogenes*. Also, immunocompetent/healthy individuals are advised to handle high-risk foods with great caution and store these foods for a relatively short time at low temperatures [142]. This is because of the severity of the diseases, the uncertain infectious dose, and the varying virulence among the strains. The strain variation in the bacterium implies not all the strains are identical, thus a prominent variation is expressed in terms of adaptation to the environment, virulence, and resistance to adverse conditions [237].

## 4. Epidemiology and Transmission of *L. monocytogenes*

Globally, the changing lifestyles, the present economic systems, a curiosity for diverse culinary dishes that are far from our traditions, and following the COVID-19 pandemic, there has been a notable rise in the consumption of ready-to-eat (RTE) foods [2]. Consequently, this positively affects the retail and food service industries. Considering health, economy, and culture, meat and its products form the greater proportion of the typical food consumed by the human population across the globe. However, the safety of meat products becomes crucial due to the initial microbial load of ingredients involved in their preparation. Microbial/biological contamination of food presents a public health menace. According to Bhatia and colleagues [238], the safety of RTE foods relies on the type of raw ingredients used and the processing time, coupled with other intrinsic parameters, including water activity and pH. Meat and its derivatives, among other animal-originating foods, need great attention as they are highly perishable, owing to their composition that represents a reliable source of a variety of nutrients, including the water activity and the optimum pH that favours the growth of microorganisms [239]. Thus, meat and its products are regarded as common primary reservoirs for microbes [240]. However, meat products, while passing through the phases of handling, processing, and storage, can further exacerbate the microbial level to a non-complaint or borderline starting situation [2]. They are consumed as both processed and unprocessed meat products. Processed meat describes meat that has been modified to fulfil one of these two conditions: firstly, to improve its taste or secondly, to extend its shelf life. The consumption of processed meat is high, especially in South Africa. This is because meat preparations are appropriate for utilisation of varying cooking techniques and therefore satisfy the consumers who prefer meat products ready to cook or eat. The increased demand for processed meat in the country is attributed to increased disposable income, an increase in the fast-paced lifestyle of consumers, convenience, and the desire to spend less time cooking. In recent times, saving time while cooking is the desire and priority of most families [241].

Nevertheless, processed and unprocessed meat products may be a threat to public health if contaminations with zoonotic pathogens, e.g., *Campylobacter* sp., *E. coli*, *Listeria monocytogenes*, etc., are found in them, since these bacteria exist as part of the intestinal flora in the animals. Therefore, the contamination of meat and the environment during the production process becomes feasible. Food-borne pathogens and their associated disease outbreaks usually take place and create constraints for consumers in the different parts of the world, leading to morbidity, mortality, and economic losses. As opposed to the diseases caused by the other food-borne pathogens, the incidence of listeriosis is rare but quite lethal to the vulnerable group, consisting of neonates, the elderly, and pregnant women, among others [242]. Among the food-borne diseases, listeriosis is one of the most severe, caused via the consumption of foods contaminated with *L. monocytogenes* [242]. Examples of the foods include soft or semi-soft cheeses, milk, undercooked and ready-to-eat foods, as well as unwashed raw vegetables and fruits.

Zakrzewski et al. [243] noted that the prevalence of *L. monocytogenes* was higher in the processed forms of foods, unlike the raw foods, as well as noting a higher level of contamination by the bacterium in developed than developing countries. Even within regions or provinces or states of a country, *L. monocytogenes* displayed variation in its prevalence. Accordingly, Ajayeoba and others [244] noted varying prevalence rates of the bacterium recovered from RTE vegetables sold among the different states in Nigeria, with incidence rates in the increasing order of 33.33% (Ekiti), 34.38% (Osun), 44.09% (Ogun), 48.75% (Oyo), 48.89% (Ondo), and 55% (Lagos). The authors highlighted that the variation in the prevalence could be ascribed to differences in farming practices and postharvest handling approaches employed by the farmers, the level of knowledge of the retail sellers, introduction to environmental pollution, and the temperatures at which the RTE vegetables are stored by the local farmers in the different states. Furthermore, Adeoye et al. [245] affirmed the existence of unequal and variable availability of vegetables from one location to another in the Nigerian markets, which is influenced mostly by the culture of the local population, socioeconomic conditions, varieties of foods, and dietary preferences. The distribution of the bacterium is not surprising among RTE vegetables, because microbial contamination is highly possible, owing to the farming and handling practices of the local farmers—for example, the continuous use of untreated wastewater and animal faeces as manures for fertilisation to improve the growth and yield of fruits and vegetables [246]. Consequently, the aforementioned foods can be referred to as vehicles of transmission to humans caused by their direct contamination [79].

### 4.1. Policy and Standard Compliance: Analysing Compliance with the Regulatory Framework Through Linking Monitoring Strategies to HACCP

Food safety in all manufactured foods could be advanced by federal regulations that provide steady methods for *Listeria* control [247]. Due to the capacity of *L. monocytogenes* to grow during storage at low temperatures, strategies to prevent contamination, reduce the number of pathogens, and limit the growth of this pathogen are very imminent [248]. Moreover, the control of contamination in the food-processing facilities based on *L. monocytogenes* is quite challenging because of the abilities of the organism to adapt to and resist conventional methods that control its presence [247]. It is noted that *L. monocytogenes* is found in the environment persisting for years; therefore, the premises of food production facilities are subjected to continuous risk from the introduction of the pathogen. The approaches to limiting *L. monocytogenes* contamination in food and addressing the risk of listeriosis largely depend on stringent hygiene and sanitary practices throughout farming, processing, and environmental management [249].

Environmental *L. monocytogenes* contamination is the primary source of contamination post-processing, and the contamination by persistent *L. monocytogenes* is incriminating in most of the contamination events occurring in the environment [250]. The bacterium has been recovered from floors, walls, and drains; thus, environmental monitoring appears as a central factor in a detailed food safety plan. An effective food safety plan is anticipated to prohibit the harbourage of pathogens in the production environment, added to reducing the possibility of cross-contamination and consequent adulteration of the final product [251]. The evaluation of the effectiveness of a company’s food safety plans is performed through the environmental monitoring programme. *L. monocytogenes* contained in the raw materials can possibly form niches in the processing environment, where it ultimately contaminates the final products. The consistent identification of *L. monocytogenes* via sampling and testing is essential in food-processing facilities over many years to facilitate the determination of persistent strains [252].

The regulatory framework consists of rules, laws, standards, and regulations established by the government agencies or industry bodies governing a specific sector that the food industry needs to abide by in order to put in check the level of contamination of RTE foods with *L. monocytogenes*. Microbiological food safety standards offer a protective and regulatory guide that guarantees the protection of the consumer against food safety hazards concerning food consumption [249]. Aligning with the standards helps companies to avoid legal repercussions and maintaining the integrity of their operations. This is because the occurrence of *L. monocytogenes* in food products produced and processed by food industries could cause a loss of finances, since the collection and control of pathogens in the food production environment is associated with costs [253]. These regulatory frameworks are very pertinent, more especially in the case of foods described as RTE foods, that are of higher risk since no further cooking or other antimicrobial interventions exist between the end of food production and the moment of ingestion by consumers [253,254]. Accordingly, Janez et al. [15] recounted that strict food regulations based on the occurrence of *L. monocytogenes* in ready-to-eat foods have been established in several countries and all strains grouped under this species are currently addressed as virulent.

The regulatory framework sets boundaries or limits to the level of the pathogen occurring in the food material by controlling *L. monocytogenes* cells and can be fulfilled by performing the hazard analysis and critical control points (HACCP) strategy in the food industry [249]. This is a cost-effective management system of choice for food safety in controlling contamination or cross-contamination of products [255]. HACCP is viewed as a crucial risk-prevention and management programme all throughout the food-processing environment, in both high- and low-risk environments. The HACCP system has been subjected to considerable study, refinement, and testing throughout the years since its introduction. The implementation of HACCP at different stages of food production will lead to the reduction in risk of food contamination [256]. This is because implementing HACCP and establishing effective critical control points in food-processing plants is eminent. HACCP is a systematic preventive system applied to address food safety through identifying, evaluating and controlling potential hazards from the biological, physical, and chemical perspectives, from raw material production, procurement, and handling, to manufacturing, distribution, and consumption of the finished products (i.e., from ingredients to process to final product), therefore preventing food-borne illnesses. It relies on the application of scientific principles to food processing and production. The fundamental goal underlying the HACCP system is to prevent problems from occurring; thus, the system embodies seven principles, including performing a hazard analysis, determining critical control points, establishing critical limits, establishing monitoring techniques, establishing corrective measures or actions, establishing verification methods, as well as establishing record-keeping and documentation methods. Specific practices are demonstrated by a particular food industry to meet the principles of HACCP because industries differ in terms of the type of facilities, the types of food product being processed, company’s policies, and/or a combination [251]. This indicates that each food safety plan is individualised per facility, as the design of the plan depends on the type of food being processed. Accordingly, Magdovitz et al. [251] emphasised that a more elaborate plan is needed for RTE foods relative to non-RTE (low-risk) foods, since no additional post-process preventive control steps are necessary with RTE foods.

A critical control point is a point, stage, or procedure at which control can be implemented to prevent, reduce, and control food safety hazards to acceptable levels based on regulatory standards. Involving such systems, the occurrence of a deviation indicates that control is lost, and the deviation is detected, but adequate steps are implemented to reestablish control timeously so that the potentially hazardous products do not meet the end users (the consumers). Monitoring of the critical control points via the measurements of critical limits is most appropriately achieved by using physical and chemical tests and visual inspection, and not by microbiological tests because of the time needed before results are obtained. Notwithstanding this, microbiological analysis is employed to verify the workability of the overall HACCP system. HACCP implementation, through which critical control points are established, is very essential [249], cutting across the aspects of farming, processing, and environmental management. Tompkin [257] emphasised that recontamination from the processing environment is viewed as the major channel of contamination of RTE with *Listeria* monocytogenes. Moreover, listeriosis is often associated with different groups of RTE foods; therefore, monitoring the environment where these RTE foods are manufactured is paramount for their microbiological safety [258]. The environmental monitoring programme involves the sampling of equipment, tools, surfaces, personnel, and facilities via microbiological techniques to detect *L. monocytogenes* so that required actions are taken to avoid contamination. The data recovered from this programme help in determining the prevalence and sources of *L. monocytogenes* in the production environment verify the effectiveness of the control measures targeting *L. monocytogenes* and locate the areas (alongside harbourage sites) and activities to improve control [259]. Authors further mentioned that the strategy for environmental monitoring involves the classification of the facility into zones 1 (food contact surfaces, e.g., utensils, conveyors, slicers, mixers, and even hands handling the food), 2 (regions immediately adjacent to food contact surfaces, e.g., equipment panel, bearings, aprons), 3 (non-food-contact surfaces within the production area, e.g., drains, floors, walls, ceilings, and pipes), and 4 (non-production area of the facility, e.g., loading dock sites, hallways, and cafeterias). Sampling should focus on high-risk areas in which a higher frequency of contamination can occur, such as zones 1 and 2 [260].

Apparently, the recovery of *L. monocytogenes* from samples collected from these zones indicates the likely contamination of the product, which will require corrective measures and even recall of the products. The identification of harbourage sites that are indicative of early contamination of the facility is achieved through detecting *L. monocytogenes* in samples collected from zones 3 and 4, before it reaches the product [261]. The environmental monitoring programme is complex and focuses on the time and frequency of sampling, the size of the sample, the methods of sample collection, along with detection methods [259]. Salza and colleagues [258] categorised the detection methods into culture-based methods and novel methods, including molecular, immunological, biosensor, spectroscopic, microfluidic system, and phage-based methods. The authors added that sample enrichment and quantitative PCR demonstrated greater sensitivity than culture-based methods while evaluating food (36) and environmental samples (254) for contamination with *L. monocytogenes* during the environmental monitoring performed across 14 dairy processing facilities. During monitoring of food-processing plants, samples of food products (raw material and final products) are equally examined through microbiological techniques to ascertain whether the concentration of the bacterium of interest complies with the quantity stipulated as standards in the regulatory framework.

The level of *L. monocytogenes* considered as tolerable for humans varies among the different countries of the world, which is guided by their individual regulatory framework. Thus, the regulations with respect to the presence of *L. monocytogenes* in food depend on the local authorities [262]. Clearly, the food codex of Turkey and the USA highlighted that there should be no detection of *L. monocytogenes* (0 CFU/g) in 25 g of an RTE sample [90,247]. Contrarily, the European Commission regulations of 2073/2005 and no. 1441/2007 emphasised a threshold limit of <100 cfu/g of *L. monocytogenes* in RTE at the time of consumption and not greater than the limit of 100 cfu/g throughout the shelf life of the RTE products [263]. In Brazil, Costa et al. [264] remarked on the present regulation for *L. monocytogenes*, which prohibits more than 10^2^ per gram or millilitres in RTE foods throughout their shelf life to comply with microbiological standards, but for infants, a zero level of the bacterium in 25 g of RTE food is recommended. The presence of *L. monocytogenes* in food is strictly prohibited by the National Food Safety Standard in the nation of China [30].

### 4.2. Prevalence of L. monocytogenes in Ready-to-Eat (RTE) Food Products and Listeriosis

The prevalence of *L. monocytogenes* is high, occurring in both clinical and food samples owing to its potential to grow and thrive in a wide range of temperatures (including refrigeration temperature) and pH levels, high salinity, low water content, and hypoxic conditions [60]. The meat and its derivatives, originating from animal sources, are of great concern for food safety and quality as they serve as major reservoirs for *L. monocytogenes*. Therefore, if meat becomes subjected to unsuitable handling processes and the time and temperature of exposure are not considered, it might render the meat as a favourable medium for the growth of pathogenic microbes in humans [2]. In food industries, plus their environment, the bacterial organisms exhibit a tendency to form biofilms that can resist standard cleaning procedures as well as the application of disinfectants [88], consequently increasing the likelihood of the occurrence of the bacterium in the vicinity of the food-processing environment and facilitating its transfer between sites and equipment, as well as to food products and humans that appear in the said areas.

Altogether, the consumption of processed ready-to-eat (RTE) food is a very significant source of *L. monocytogenes* infection. Clearly, major large listeriosis outbreaks were associated with RTE foods that have a long shelf life, stored at low temperatures until consumption, alongside being consumed without further cooking or treatment, thereby encouraging the growth of *L. monocytogenes* [265] and serving as suitable media for the growth of the bacterium as well as allowing it to remain viable [223]. The ability of *L. monocytogenes* to thrive at refrigeration temperature in foods with a reasonably low moisture content and high salt concentration causes it to persist and multiply, rendering it difficult to control in the food environment [266]. Several cases of listeriosis originating from the consumption of RTE food have been reported across the globe. The disease occurrence is sporadic but could result in severe damage during an outbreak [267]. There appears to be a variation in the prevalence, which could be explained in part by the regulations governing the acceptable microbiological level or threshold limit of *L. monocytogenes* in RTE foods in different countries.

In addition, the type of RTE meat product is of great consideration as the assorted products have different methods of preparation, leading to varying lengths of shelf lives (spanning 23 to 30 days for sausages and 60 days for diced cooked ham) that favour the growth of *L. monocytogenes* [268]. Most interesting were the findings of Kurpas and colleagues [269], who noted varying prevalence rates of *L. monocytogenes* in the same type of sample in different countries. Notwithstanding this, Castrica et al. [2] affirmed that the differences in the prevalence rates of the said bacterium could reflect how large retailers, restaurants, or canteens execute their own good hygiene practices during the preparation, handling, processing, and storage of the RTE foods.

#### 4.2.1. Developing Countries

*L. monocytogenes* is a subject of great interest and studies in the meat industry; however, it is of a relatively low priority inpublic health systems in African countries. This is because listeriosis is less frequently reported as opposed to salmonellosis, campylobacteriosis, and *E. coli* gastroenteritis in African countries [244], as these countries offer great attention to food security and major health challenges, including malaria, HIV/AIDS, and tuberculosis [242]. Historically, listeriosis is of low incidence in African countries. Accordingly, Barrett and colleagues [270] revealed that low-income countries embrace rural value chains (traditional food value chains) constituting foods produced and consumed at home, that are limited in diversity but dominated by staple grains and a few processed animal products. Although these rural agri-food systems are exposed to poor hygiene and sanitation conditions alongside very close interactions with farm animals, heightening the perils of contamination of food with *L. monocytogenes* as well as creating huge chances for direct human exposure, the home cooking of foods without extended storage of the foods in the refrigerator averts the excessive multiplication of the psychrotolerant bacterium to hazardous levels, thus lessening the likelihood of contamination [271].

Contrary to the historical data, the largest outbreak was observed in South Africa (a middle-income country) between 2017 and 2018, wherein 216 people of the 1060 confirmed laboratory cases died [272]. This event of a such magnitude could mirror the weaknesses of food safety systems and the lack of knowledge of the risk parameters influencing the dynamics of transmission of *L. monocytogenes* in the African food value chains [242]. As a consequence of the outbreak, South Africa is the only country that has categorised listeriosis as a notifiable condition [273]. The transition from a low- to a middle- or higher-income status of a country’s economy often provokes the transformation of the food safety system, resulting in diversification of diets and the choice of foods by consumers. In this light, Milford and coauthors [274] noted the increased meat consumption among the South African population and ascribed the rising level to rapid population growth, elevated income, and rising urbanisation. Therefore, food value chains experience changes from traditional through to transitional and modern, as the population tends to consume more animal-derived foods, added to processed RTE foods, because of their nutritional benefits and convenience. This changed lifestyle disproportionately amplifies the risk of exposure of the population to *L. monocytogenes* [270,275].

In the said country, the food value chains are in the transitional stage, dominated by informal wet markets, street vendors, and the presence of midstream, small–medium processors, leading to increases in the diversity of foods to encompass fresh animal-originated foods (dairy, fish, poultry, and beef) that are perishable, as well as horticultural commodities that need preservation and refrigeration [276]. Barrett and coauthors [277] highlighted that the concentrated purchasing power of the urban populations leads to a demand for high-value processed, ready-to-cook, and RTE food that salvages the waste of time for consumers. Steyn et al. [278] noted that about 30% of the South African population residing in urban communities depend on foods sold by vendors on the streets to meet their daily dietary needs. Apparently, RTE foods sold on the streets for immediate consumption are a popular trait of the informal markets that occur in most African countries, including South Africa. However, the safety of such foods is compromised, subjected to unintended cross-contamination originating from poor handling practices, absence of running water, washing, and disinfection facilities [279]. According to Borena et al. [79], the safety of meat products relating to food-borne diseases in developing countries is an issue of great significance. This is due to poor practices and the production of meat and its products under unhygienic conditions. In African middle-income countries, including South Africa, food value chains are likely associated with a heavy burden of food safety challenges as the combined effect of elevated ambient conditions of the African tropical climate, the poor refrigeration or the lack thereof, alongside the protracted storage of foods following preparation heightens the menace of bacterial growth after the food is contaminated [280].

In an African setting, several risk factors are known to exacerbate consumers’ exposure to *L. monocytogenes* and infections, and these include: the consumption of high-risk RTE foods, the challenges in cold chain (refrigeration), the effects of climate changes, deficiency in food safety knowledge and practices of informal food vendors, the regional food trade and globalisation, and the population at risk [242]. Overall, in developing countries, street-vended foods are regarded as a major threat to public health owing to the lack of elementary infrastructure and facilities to uphold their mobility, diversity, and temporary nature, which could drive or propel contamination microbes from sand, water, dust, and the air [281].

#### 4.2.2. Developed Countries

In the said countries, Anonymous [282] and Lukinmaa et al. [283] noted that the cases of listeriosis stood at 0.2 to 0.8 cases per 1000 persons a year, suggesting 1600 to 8400 cases with 320 to 2500 deaths per year in Europe. However, the authors expressed that the wide range incidences may originate either from variations in the notification systems or because of outbreaks, which may cause a great increase in the number of cases (even though the exact number of cases related to the outbreak is not known, but it is usually higher than the reported figure) [283]. Seemingly, the Centers for Disease Control and Prevention [284] reported that 1600 cases of listeriosis were observed in humans in the United States yearly, resulting in a case-fatality rate of approximately 21%, as it is said to be the third-leading cause of death among the people in the country. Similarly, Khan and coauthors [237] expressed a mortality rate of 20–30% linked with *L. monocytogenes*, which was identified and tiered in the top position amongst other food-borne pathogens, as the cause of the most deaths in Wales and England by the Food Standards Agency.

Table 5 below gives the prevalence rate of *L. monocytogenes* from varieties of food samples, from animal origin as well as from RTE foods. Clearly, animal-derived protein is of great importance to humans to help maintain the normal functioning of their bodies [285]. The demands of modern life and current feeding habits favour the development and availability of certain food types that require no further heating or cooking prior to consumption, or ready-to-eat foods, which are more likely to be associated with listeriosis outbreaks. It is apparent that food contaminated with *L. monocytogenes* remains a problem in both developing and developed countries [269]. Across the globe, as one transits from one country to the next, the presence in terms of prevalence of *L. monocytogenes* can be determined through different methods, with all exploiting the characteristics (phenotypic, genotypic, and growth properties) of the organism. The prevalence is also not static among the different food types and has an impact on both the positive predictive value and the negative predictive value of tests [286]. Very interesting among the findings are the information/data presented by Marina et al. [287] and Gyurova et al. [288], relating the condition of *L. monocytogenes* in peninsular Malaysia and Bulgaria, respectively. Marina et al. [287] reported a zero (0) percent (0%) prevalence of *L. monocytogenes* in their analysed samples, comprising dairy milk and meat products, and ascribed such results to the compliance of the food-processing plants to the standards of hygiene and sanitation and quality assurance, in addition to food safety systems. Similarly, Gyurova and colleagues [288] in their study noted a zero percent (0%) prevalence of *L. monocytogenes* in their RTE heat-treated meat and soft and semi-soft cheese procured from four cities in Bulgaria. The authors further emphasised that they strictly followed the procedures designed for receiving and processing samples, which, in my opinion, gave no opportunity for contamination with the pathogen.

Nevertheless, discrepancies in the prevalence rates are observed, which could be associated with the different methodologies employed for isolation, lack of physical separation between the raw and cooked food areas (cross-contamination), the country of investigation, insufficient heat treatment, the level of hygiene practised in the meat-processing factories, slaughterhouses, and butcher shops, the season of the study [268], meat storage conditions, and sample size, added to the different food types, as meat products vary in terms of matrix (affecting the level of contamination) [268,289], and deficiencies in manufacturing in terms of processing, finishing, packaging, product storage, distribution, marketing storage, and commercial refrigeration [242]. In addition, the lack of a similarly strict regulatory framework for the retail and food service sector (restaurants, grocery stores, delicatessens, etc.) and the relatively scarce application of antimicrobials may affect the occurrence or transmission of *L. monocytogenes* to these food types [48]. Food manufacturers are more liable to employ the hazard analysis and critical control points (HACCP) system and offer training and food hygiene courses for food handlers in an attempt to control the presence and or transmission of *L. monocytogenes* in their premises or environment [251,290]. On the other hand, retailers and caterers, owing to the unique characteristics of their environments, including complexity, variety, and a highly dynamic approach to operation (e.g., such premises are open to the public, require them to display, slice, and repackage some products, and have a high level of dependence on temporary food handles, added to the associated high employee turnover) may prevent the development and application of suitable control measures. Accordingly, the changing values of contamination prevalence and levels registered in the different countries by the various researchers mostly mirror corresponding geographic differences and differences in regulatory policies and control measures employed in the different countries [291].

The prevalence of *L. monocytogenes*, as presented in Table 5, is a proportion denoting the percentage of the investigated food samples harbouring the organism at that given time. The values were calculated by dividing the number of positive tested samples by the total number of food samples involved in the investigation [286]. According to Tirloni and colleagues [292], the prevalence rates need to be carefully considered because they highlight the exposure of the public to the presence of the pathogen on a frequent basis; the potential circulation of the pathogen within the indicated regions/countries, and the need to adopt appropriate intervention measures at the sample sites. The prevalence of the pathogen indicates the effects of its risks and disease stress on the population [293]. The presence of the pathogen in RTE meat and pasteurised milk and cheese is a cause for concern, though conditions for its growth are not favourable. This is because some cells may survive in adequate numbers to cause infection in susceptible individuals. *L. monocytogenes* cells have the potential for long-term survival based on the type of product under refrigeration conditions, therefore increasing the chances of transmission [237].

Regardless of the measured value (whether as low as 0% or high as 50%), these foods derived from animal origin will serve as vehicles for transmission of the opportunistic pathogen to other products that encourage the growth of the pathogens and to humans, which might provoke the manifestation of the infection listeriosis as well as create a possibility for an outbreak [291]. This, however, depends on the population that is infected as well as the viable counts of the organism tested. The vulnerable population (relating to the state of the immune system) requires a lower infectious dose of the pathogen than the healthy population to trigger disease outcomes, either febrile gastroenteritis or invasive listeriosis caused by the pathogen [294]. Considering that *L. monocytogenes* demonstrates strain divergence (in terms of virulence traits), the severity of the disease outcome can also be influenced by this feature. Even though strain divergence is among the parameters affecting the infectious dose of *L. monocytogenes*, currently, all strains of this bacterium are considered potentially pathogenic from a food safety perspective. Table 5 harbours valuable epidemiological data that narrate the contamination level of different RTE foods and animal-derived products in the different countries of the world, thus suggesting that *L. monocytogenes* is highly prevalent in these categories of foods in some countries across the world [266]. These data equally translate to the food safety issues pertaining to *L. monocytogenes* of these tested food products. Food safety remains an ongoing topic, and it is most critical in terms of the group of people with an increased risk, such as infants, the elderly, the immunocompromised, as well as pregnant women [243]. According to Marina and colleagues [287], food safety assurance systems play a crucial role in the execution of the nation’s food safety programmes and policy. Therefore, the data generated through these findings underscore the necessity to strengthen communication between public health and food surveillance [264].

Taking into consideration Table 5, it is observed that most of the foods responsible for the outbreak of listeriosis were equally cited in Table 5. Emphatically, Meloni [295] mentioned that *L. monocytogenes* has the capacity to reproduce at varying rates during storage at refrigeration temperatures, based on the type of food, both under aerobic and anaerobic conditions, its response to disinfectants, and the ability to adhere/attach to different surfaces even when it occurs in small numbers in the food type originally. The occurrence of this bacterium in these RTE foods is a cause for concern because of the potential of the bacterium to multiply in several foods at low temperatures, ranging from 2 to 4 °C. [296]. Morshdy et al. [285] elaborated that workers’ hands, clothing, knives, cutting boards, meat-processing plant environments, and slaughterhouses are among the factors influencing the microbiological conditions of meat. In these studies performed as presented in Table 5, most of these food sources were procured from retail shops, streets, open-air markets, butcheries, and restaurants from which *L. monocytogenes* isolates were recovered, thus indicating a breach of quality assurance [297].

The organism can be diagnosed using both classical microbiology and molecular-based methods, which could lead to the determination of the prevalence rate of *L. monocytogenes* [201]. Knowledge of the level and the prevalence of the bacterium in various foods may be useful for policymakers, medical professionals, and health units to use the data to inform testing parameters for surveillance and to educate immunocompromised persons about their risk of acquiring listeriosis, as well as health inspectors and the general population about high-risk foods [202].

**Table 5:** Prevalence of *Listeria monocytogenes* in animal-originated and RTE foods. Listeriosis outbreaks across the globe, the RTE foods involved, and the number of deaths registered.

Food Samples	Prevalence Rates (%)	Methods Used for the Recovery of *L. monocytogenes* from Samples		Regions/Countries	References
384 RTE foods consisting of raw and pasteurized milk, cheese, cream cakes, ice cream, minced meat, pizza, and fish	6.2	Fraser broth + ferric ammonium citrate as supplement (primary and secondary enrichment with incubation at 30 °C for 24 h and 37 °C for 48 h, respectively). Polymixin acriflavine Lithium Chloride ceftazidime Aesculin (PALCAM) (cultivation and isolation). Confirmation obtained by transferring presumed *Listeria* sp onto tryptone soya yeast extract agar, incubation at 37 °C for 24 h. Further confirmation by Gram staining, haemolysis, motility, catalase, CAMP test, and sugar fermentation.		Gondar Town (Egypt)	Garedew et al. [87]
250 chicken carcasses	9.6	Primary and secondary enrichment performed in Fraser broth, and both incubations carried out at 30 °C for 24 h. Fraser broth cultures were plated on PALCAM, incubated at 37 °C for 48 h. Confirmation via catalase, oxidase, and sugar fermentation tests plus haemolysis type. Isolates further verified API *Listeria* test (BioMerieux).		Mansoura city (Egypt)	Zakaira and Sabala [97]
90 chicken and beef	45	Samples homogenised in 0.1% buffered peptone water and serially diluted; 1 mL serial dilution inoculated onto *Listeria* CHROM agar and incubated at 37 °C for 24 h. Blue colonies with halos were confirmed using Gram staining. Confirmed colonies were transferred into Brain Heart Infusion broth, incubated at 37 °C for 24 h, and further confirmed via polymerase chain reaction.		Zanjan city (Iran)	Farhoumand et al. [98]
65 raw milk, pasteurized/fresh milk, cheese	18.46	Primary and secondary enrichment of samples were performed in Fraser broth based with Frazer selective supplement (SR0166E, Oxoid, UK) of different strengths. Initial incubation at 30 °C for 24 h and secondary at 37 °C for 24–48 h. Broth cultures were plated on chromogenic *Listeria* agar (ISO) base mixed with OCLA (ISO) differential (SR 0244E) and selective (SR 0226E) supplement in addition to Brilliance *Listeria* Agar Base, seeded with Brilliance differential (SR0228E) and selective (SR0227E) supplements. All supplements procured from Oxoid, Ltd., UK and incubation carried out at 37 °C for 24–48 h.		Amathole, Chris Hani and Sarah Baartman District Municipalities, Eastern Cape Province (South Africa)	Kayode and Okoh [96]
100 raw kebab and hamburger (RTE Food)	50% and 22%, respectively	Samples were enriched in *Listeria* enrichment broth and incubated at 30 °C for 4 h. *Listeria* selective enrichment supplement was introduced into the broth and incubated further for 44 h. A loopful of the enrichment broth was plated onto PALCAM *Listeria* selective agar and incubated for 48 h at 35 °C. Suspected colonies were confirmed via Gram staining, motility, catalase, urea, haemolysis, CAMP, and sugar fermentation tests.		Tabriz city (Iran)	Rajaei et al. [104]
750 meats (chicken, chevon, pork, and beef), milk, and milk products (curd and paneer)	6.4	Fixed quantity of samples mixed with PALCAM broth plus *Listeria* supplement and incubated for 24 h at 30 °C. Broth culture (loopful) streaked onto PALCAM agar for 24 h at 30 °C. Suspected *Listeria* colonies were confirmed preliminarily using Gram staining, catalase, oxidase tests. Typical colonies of *Listeria* were transferred into BHI broth and incubated at 25 °C overnight. Motility and catalase-positive and oxidase-negative isolates are further confirmed, biochemically and by PCR.		Guwahati (India)	Deka et al. [298]
30,016 RTE foods (meat, fish, culinary, pastry, fruit and vegetable products, gravy sauce, mixed salads, and meals)	3.6	Primary and secondary enrichment in both half- and full-strength Fraser broth. Initial incubation for 24 hat 30 °C and 37 °C for 48 h. Both broth cultures were plated on ALOA and PALCAM agar and incubated at 37 °C for 24–48 h. Presumed *L. monocytogenes* colonies were streaked on tryptone soya yeast extract agar and incubated a 37 °C for 24 h. Confirmation test via catalase test, Gram staining, microscopy, motility, CAMP tests, and sugar fermentation.		Estonia	Koskar et al. [299]
1000 RTE foods (bacon, chorizo paisa, grilled hamburger meat, mortadella, and salami)	16.3	ISO 11290-1. A portion of sample was homogenised in half-strength Fraser broth and incubated at 30 °C for 24 h for primary enrichment. Secondary enrichment was performed in full-strength Fraser broth and incubated for 24 h at 37 °C. *L. monocytogenes* colonies were isolated and identified on *Listeria* chromogenic agar.		Quevedo (Ecuador)	Meta-Bone et al. [268]
132 RTE seafoods (smoked fish, salted fish, dried fish, raw marinated fish, cooked marinated cephalopods, surimi crab sticks)	6.1	ISO 11290-1/A1 procedure was employed. A fixed portion of sample was homogenized in half-strength Fraser broth and incubated at 20 °C for 1 h to recover stress organisms. For primary enrichment, the homogenate was supplemented with Fraser half-selective supplements and incubated for 24 h at 30 °C. An aliquot of primary enrichment culture was transferred into Fraser broth and incubated for 48 h at 30 °C for secondary enrichment. Subsequently, both primary and secondary broth cultures (a loopful) were streaked onto ALOA and Oxford Agar and examined for growth after 24 and 48 h at 37 °C. Suspected colonies were purified by growing on tryptone soya yeast extract agar. Pure cultures were subjected to multiplex PCR for bacterial identification.		Thessaloniki (Northern Greece)	Soultos et al. [263]
6000 poultry, pork, and beef	2.1	technique was employed. For primary enrichment, sponges obtained from samples were dipped in half-strength Fraser broth and incubated for 24 h at 30 °C. Sponge was squeezed firmly multiple times into the bag and secondary enrichment performed in full-strength Fraser broth; incubation occurred at 37 °C for 48 h. Reductive inoculation of cultures was carried out on Chromocult *Listeria* Selective Agar (ALOA) and incubated at 37 °C for 24 h. Suspected colonies were transferred to Columbia agar with 5% sheep blood. Confirmatory tests included haemolysis type and polymerase chain reaction.		Poland	Skowron et al. [289]
184 meat samples (chicken, quail, duck, turkey, and pork)	10.32	Primary enrichment of samples was performed in half-strength Fraser broth and incubated at 30 °C for 24 h. Subsequently, secondary enrichment was completed in Fraser broth and incubated at 37 °C for 24 h. Cultures plated on ALOA and incubated for 24–48 h at 37 °C. Suspected colonies were purified in tryptone soy agar and BHI broth. Purified isolates were identified via matrix-assisted laser desorption/ionization–time of flight Mass spectrometry (MALDI-TOF MS) Biotyper.		La Rioja (Spain)	Martinez-Laorden et al. [300]
200 minced meat, poultry meat, tilapia fish, and raw milk	10	Enrichment was carried out in both half- and full-strength Fraser broth and plated on Oxford and Agar *Listeria* Ottaviani and Agosti (ALOA) agar. Colonies were purified on tryptone soya yeast extract agar and suspected colonies were confirmed by beta-haemolysis, triple iron sugar, and oxidase tests. Further confirmation by multiplex PCR.		Sharkia Province (Egypt)	EL-Demerdash and Raslan [297]
1096 (dairy products, bovine meat products, pastry, salads, poultry meat products, chickpeas cooked with eggs, and mayonnaise	1.5	Enrichment steps and selective media were employed. Fraser broth was used to grow the bacterium, while plating was carried out on selective media (e.g., ALOA). Isolates were identified biochemically using API-*Listeria* system and PCR serogrouping.		Tetouan, North-Western (Morocco)	Amajoud et al. [301]
443 pasteurised milk, raw milk, and yoghurt	5.6	Enrichment of samples was performed in *Listeria* broth-LEB and incubated for 4 h and medium later, seeded with *Listeria* selective supplements, and further incubated for 48 h at 30 °C. Bacterial growth was streaked on Oxford Agar plates following 24 and 48 h of incubation and maintained at 35 °C. Suspected colonies were transferred into tryptic soy yeast extract agar and incubated at 30 °C for 24 to 48 h. Confirmatory tests included microscopy, catalase, sugar fermentation, and CAMP tests.		Addis Ababa, Ethiopia	Seyoum et al. [302]
400 raw beef, RTE food products, milled beef, offal, and organs	8.3	Meat sample homogenized in ONE Broth-*Listeria* and incubated at 35 °C for 48 h. Enriched broth samples inoculated onto Chromogenic Brilliance *Listeria* agar and incubated at 35 °C for 48 h. Suspected colonies identified using phenotypic and molecular methods.		Mpumalanga (South Africa)	Moabelo et al. [303]
567 retailed raw foods (fishery products, raw/fresh meat, frozen food, edible fungi, and vegetables	22	The National Food Safety Standard of China—Food microbiological examination: *L. monocytogenes*. Samples were homogenized in *Listeria* enrichment broth I (LBI) and incubated for 24 h at 30 °C. Secondary enrichment was carried out in LB2 and incubated for 24 h at 30 °C. Enrichment broth (LB2) culture (a loopful) was inoculated onto Chromagar *Listeria* selective agar plates and incubated for 48 h at 37 °C. Suspected colonies were identified via the Microgen ID *Listeria* identification system.		Guangzhou city (South China)	Chen et al. [304]
3171 food samples (frozen, deli meat, RTE, and cheese.	11.2	Primary and secondary enrichment conducted as above in half and full strength fraser broth. Real-time PCR Dupont Qualicon BAX^R^ system was employed. Positive real-time PCR samples were streaked on Oxford medium base with modified Oxford antimicrobic supplement and on BBL^TM^ CHROM agar ^TM^ *Listeria* (BD) and both incubated at 35–37 °C for 24–48 h. Suspected colonies were identified using API *Listeria* kit, beta-haemolysis, halo production, catalase reaction, bile esculin, and Christie–Atkins–Munch–Petersen (CAMP) test.		Montevidio city (Uruguay)	Braga et al. [265]
122 RTE smoked and gravad fish (retailed products) 63 RTE soft and semi-soft cheese and 60 heat-treated meat products	12.3 0	A fixed portion of sample was homogenized in half-strength Fraser broth and incubated at 20 °C for 1 h to recover stress organisms. For primary enrichment, the homogenate was supplemented with Fraser half-selective supplements and incubated for 24 h at 30 °C. An aliquot of primary enrichment culture was transferred into Fraser broth and incubated for 48 h at 30 °C for secondary enrichment. Subsequently, both primary and secondary broth cultures (a loopful) were streaked onto ALOA and Oxford Agar and examined for growth after 24 and 48 h at 37 °C.		Bulgaria	Gyurova et al. [288]
130 dairy milk products (ice cream, butter, and cheese) and meat products (chicken, frankfurter, smoked chicken frankfurter, chicken sandwiches, and chicken lyoner)	0	Primary enrichment of 25 g of sample in University of Vermont (UVM) broth (225 mL, i.e., 1:10 dilution) and stomached for close to 2 min. This was incubated at 28–32 °C for 20–26 h. A total of 100 µL of UVM was dispensed into 10 mL of Fraser broth for further incubation performed at 33–37 °C for 18–24 h. Inoculum was plated on chromogenic selective agar, ALOA, and incubated at 33–37 °C for 2 days. ALOA agar plates were assessed for typical *L. monocytogenes* colonies (blue-green colonies with a halo).		Peninsular Malaysia	Marina et al. [287]
132 RTE delicatesses (vegetables with mayonnaise sauce, starts without mayonnaise, pasta/rice-based, meat-based and fruit-based courses.)	17.4	25 g of product was diluted with 225 mL of ½ Fraser broth, homogenised for 1 min in a stomacher and incubated at 30 °C for 48 h. Later, 100 µL of the homogenate was plated on Rapid L’monoagar and incubated at 37 °C for 24–48 h according to AFNOR BRD07-0405-09/98 method. In parallel, 1 mL was equally added to 9 mL of whole Fraser broth and incubated at 37 °C for 24 h. Afterwards, 100 µL of the broth was freshly streaked onto Rapid L’monoagar and incubated under the same conditions. One or two presumptive colonies were selected and subsequently identified by Microbact ^TM^ *Listeria* 12L kit.		Northern Italy	Tirloni et al. [292]
881 RTE meat, fish, and seafood products and RTE milk products	8.4	Standard microbiological and enzyme-linked fluorescent immunoassay analysed the presence of *L. monocytogenes*. A fixed portion (25 g) of test sample was enriched in ½ Fraser broth and incubated for 23 or 26 h. Secondary enrichment was performed in full-strength Fraser broth and incubated at 22 or 26 h. Inoculum of Fraser and ½ Fraser broths were streaked on selective agar and incubated for a maximum of 72 h. Incubated plates were refrigerated for a maximum of 2 days before reading. Confirmation was conducted through immunoassay carried out by automated mini VIDAS system using VIDAS^R^ LMX test kit.		Serbia	Branka et al. [305]
133 retail RTE food, including dairy, meat-based, poultry-based, vegetable-based, fruit-based, and fish-based products. RTE foods were unpackaged while others were packaged and non-frozen. All samples except the vegetable and fruit-based products were heat treated	12.8	Two-stage enrichment was carried out for the detection of *L. monocytogenes* according to the International Organisation for Standardisation standard (ISO) protocol. Each sample (25 g) was added to ½ Fraser broth (225 mL) and homogenised in a stomacher for 3 min. Homogenate was incubated at 29 or 30 °C for 22 or 24 h (primary enrichment). A total of 100 µL of the primary enrichment culture was dispensed into 10 mL of full-strength Fraser broth. The mixture was incubated at 37 °C for 40 or 48 h. A loopful of both the ½ and Fraser broth were streaked on both ALOA and PALCAM agar. Plates were incubated at 37 °C for 24–48 h. Five suspected colonies from both cultured media were picked for identification of the organism via Gram staining, catalase, oxidase, and sugar utilisation tests, CAMP test, motility at 20 °C -25 °C. Confirmation of *L. monocytogenes* isolates was performed by PCR.		Ankara city (Turkey)	Sentürk et al. [223]
**RTE Foods as vehicles**	**Year of listeriosis outbreak**	**Human cases involved**	**Deaths**	**Countries**	**References**
Bologna-style sausage (polony)	2018	937	216	South Africa	Thomas et al. [272].
Turkey meat products	2012–2016	26	3	Czech Republic	Gelbícová et al. [306].
Rillettes (pâte-like meat product) Jellied pork tongue	1999 2000	10 32	3 5	France	De Valk et al. [307].
Cheeses, a sour milk curd termed Quargel	June 2009–Jan. 2010 Dec 2009–Feb. 2010	14 20	5 3	Austria, Germany, and Czech Republic	Fretz et al. [308].
Chocolate milk	1994	45	0	USA (Illinois)	Dalton et al. [309].
Butter from pasteurized milk	1999	25	6	Finland	Lyytikäinen et al. [310].
Milkshakes from ice cream	2015	10	0	USA	Pouillot et al. [233].
Unpasteurised milk Pasteurized milk	2007–2008	449 174	5 24	Canada and USA	Sebastianski et al. [311].
Pasteurised milk	1983	49	14	USA (Massachusettes)	Fleming et al. [312]; James et al. [313].
Cheese	2007	5	3	USA (California)	Centre for Disease Control [314].
Unpasteurised chocolate milk	2014	2	1	USA (California and Florida)	Nichols et al. [315].
Cheese from pasteurised milk	2006–2007	189	0	Germany	Koch et al. [316].
Pasteurised ice cream	2014	2	1	USA (Washington)	Rietberg et al. [153].
Cheese dairy	2018–2020	79	10	Switzerland	Nüesch-Inderbinen et al. [317].
Pasteurised chocolate milk	2015–2016	34	4	Canada (Onatrio)	Hanson et al. [318].
Deli meats	2024	61	10	USA (Illinois, Virginia, New York, New Jersey, South Carolina, Florida, New Mexico, and Tennessee	CDC [319].
Fish products (RTE cold-smoked salmon, cream cod, mackerel, herrings, fish-meat product, and fish salad)	2012–2024	73	14	Czehia, Germany, Finland, Italy, the Netherlands, United Kingdom, and Belgium	ECDC, EFSA [320].
Packaged leafy green salads	2015–2016	33	5	USA and Canada	Self [321].

From Table 5 above, we can see that the listeriosis outbreaks in different countries were associated with different RTE foods and the number of persons involved, as well as the number that resulted in death varying from one region or country to the other. This observation could be attributed to the variation in hygiene, food content, and the rates of environmental contamination in the different areas [322]. The recent changes in the prevalence rate of the listeriosis outbreak could be attributed to changes in the production, processing and distribution of food, alteration in the feeding habits tailored toward RTE foods, and the aggravated use of refrigerators for food storage, added to the drastically elevated number of individuals described as high risk for the infection [323]. Even though several outbreaks have happened in developed countries, the greatest impact was felt in developing countries (e.g., South Africa). This can be attributed to the lack of resources to enforce food safety regulations, perform effective surveillance, the implementation of educational programmes focused on food hygiene, inaccessibility to potable water for drinking and food preparation, and the lack of proper means of food transportation, preservation, and storage, added to the lack of awareness about safe food handling practices [249].

#### 4.2.3. Some Specific Food Case Studies Involving *L. monocytogenes*

Firstly, Mohapatra and colleagues [324] remarked that the recurrence of *Listeria* outbreaks in the USA is a continuing issue in public health care. Datta and Burall [325] noted that approximately 1600 listeriosis cases are reported each year in the USA, associated with greater than 95 hospitalisations and between 15 and 20% deaths. TheCenters for Disease Control and Prevention [319] reported an outbreak of listeriosis involving 61 cases, caused by the consumption of deli meats sliced at various supermarkets and grocery store delis and affecting eleven (11) states in the US. The outbreak spanned from 29 May 2024, to 21 November 2024, during which 60 individuals were hospitalised and 10 persons died from eight (8) states, including Illinois, South Carolina, Virginia, Tennessee, New Jersey, New York, Florida, and New Mexico. Following interviews with some of the people infected carried out by the state and local public health officials, the following demographic information was recovered and presented; thus, age ranged from 32 to 95 years, comprising 51% males and 49% females, and of White (78%), African American/Black (16%), Asian (3%), and other (3%) descent. Ethnicity was grouped into non-Hispanic (94%) and Hispanic (6%). Among those interviewed, 27 mentioned that they consumed a variety of meats sliced at deli counters, e.g., liverwurst, while 20 individuals emphasised consuming Boar’s Head Brand in the month prior to becoming sick. According to the CDC [284], a thorough food history alongside the collection and testing of food samples from case patients navigated to the sources of contamination. Among those who died was an infant, and this prompted the recall of all RTE meat products manufactured by Yu Shang Food Inc [319]. The authors equally advised that the populations of pregnant women those aged 65 or older, or with weakened immune systems should keep away from eating deli meats, or if they really want to consume the specified food, it must be heated to an internal temperature of 74 °C or until steaming hot to destroy any microbes.

Secondly, dairy products can be contaminated following pasteurisation and are implicated in the cause of the listeriosis outbreaks. Pasteurisation is a thermal process employed in eliminating microbial contaminants in food including dairy products. However, Calahorrano-Moreno et al. [326] demonstrated a contrary finding relating to the efficacy of this method in eliminating contaminants. In this light, Medjahdi and coauthors [327] explained the risks for contamination and recontamination of pasteurised milk, which are regarded as the primary causes of early spoilage of milk today. Of more serious concern are the findings of Londoňo-Carmona et al. [328], which highlighted the presence of microbial contaminants and antimicrobial residues in pasteurised milk intended for commercial purposes and human consumption. Hanson and colleagues [318] investigated a listeriosis outbreak in Ontario, Canada over a seven-month period, from November 2015–June 2016. Source tracking identified pasteurised chocolate milk as the source. A total of thirty-four (34) case patients were involved who were identified as Ontario residents, with thirty-two (32) registered hospitalisations and four (4) deaths. Among the patients with ages ranging from less than 1 to 90 years, 59% were females. Local health professionals in Ontario completed the national invasive listeriosis questionnaires, collected food samples, performed interviews, as well as reviewed purchase records collected through shoppers’ loyalty card programs in a bid to trace the source of the outbreak. In addition, pulse field gel electrophoresis (PFGE) and whole genome (WGS) sequencing molecular subtyping approaches were conducted at the Public Health Ontario Laboratory, in accordance with PulseNet Canada protocols (Table; Appendix Figure, https://wwwnc.cdc.gov/EID/article/25/3/18-0742-App1.pdf accessed on 26 February 2025) to enable identification by the *L. monocytogenes* pathogen. According to Nüesch-Inderbinen et al. [317], it was revealed that WGS plays a key role in demonstrating close relatedness between the isolates from food, environment, and patients. The Canadian Food Inspection Agency and the Public Health Ontario Laboratory performed laboratory analyses of the food samples. The source of contamination was traced to an expired bagged chocolate milk recovered from the house of one of the patients since *L. monocytogenes* was isolated, and the PFGE pattern conformed to that of the outbreak strain.

It is obvious that following the identification of the brand, a recall of all the branded chocolate milk produced from that facility was immediately initiated because the pathogen can be transmitted to other products via contact with the contaminated products, food handlers, etc. [237]. This further prompted the sampling of the environment at the manufacturer’s site [317], which revealed the presence of the outbreak strain around a post-pasteurisation pump devoted to chocolate milk and on non-food contact surfaces [318]. Notwithstanding this, the authors further explained that the contamination could be due to harbourage sites created through a specific maintenance event or poorly designed equipment. Gupta and Adhikari [259] described harbourage sites as niches or locations within the facility or equipment that cannot be reached during cleaning and sanitisation and which food units and microbes tend to occupy. Thus, the microbes tend to persist in these sites, but *L. monocytogenes* persistence in these sites is determined by the efficacy of the cleaning and sanitation process and the concentration of cells before and following cleaning and sanitation [145]. So then, Hanson and colleagues [318] advised that careful attention should be targeted to equipment design and maintenance programmes, because harbourage sites could bring about recurring contamination that could remain unnoticed by routine monitoring. To prevent further contamination, the equipment was replaced, and corrective measures deployed to avoid recurrence. In addition, the production of chocolate milk was resumed only after thorough testing of *L. monocytogenes* was performed under regulatory supervision [318].

Thirdly and more interestingly, Self and colleagues [321] reported a multistate outbreak of listeriosis in the USA (Connecticut, Massachusetts, Michigan, Montana, New York, Ohio, and Pennsylvania) and Canada (Ontario, Quebec, Prince Edward Island, Newfoundland, New Brunswick, and Labrador) caused by the consumption of packaged leafy green salads. Involved in the listeriosis outbreak were 19 case patients with ages ranging from 3 to 83 years, constituting 74% females and one pregnant woman, who were all hospitalised in the United States of America. However, one (1) patient died. While in Canada, fourteen (14) case patients were involved and three (3) patients eventually died. According to Niu and coauthors [329], the use of a combined methodology consisting of retail target sampling, epidemiology, and laboratory data can enable the successful identification of the source of infection (packaged leafy green salads). The PFGE aided in matching the PFGE patterns of 10 patient samples obtained in Canada to the PFGE of the primary outbreak strain in the USA. Clearly, PFGE presented a conventional typing scheme that facilitated interagency communication. Notwithstanding this, wgsMLST served as a powerful tool offering greater specificity than PFGE by displaying the close genetic relatedness between the clinical isolates and those from packaged leafy green salads; thereby, it acted in distinguishing the isolates in the cluster from other *L. monocytogenes* isolates that were grouped under a common PFGE pattern but were previously isolated from multiple foods [329,330].

Following inspection of the facility, it was observed that the sampling plan of the facility based on the environment could have caused the facility not to identify contamination with *L. monocytogenes* or harbourage, which could have been responsible for the food contamination. Interventions were implemented to avoid further cases. As a preventive and control measure, the facility halted production for 4 months, allowing time for the facility to conduct testing and root cause analysis (Dole Food Company, Inc.) The company recalled the products, which presented a large economic burden to the industry and the cost of the suspended operations was estimated to be 25.5 million dollars. This was further worsened by the damage to the brand [160]. Self and colleagues [321] emphasised that the collaboration between the investigators in the US and Canada consolidated the analysis of the outbreak, which led to a timelier and detailed withdrawal and recall of products from the markets. The aforementioned case studies highlight the position of food safety risk monitoring in identifying the source of infection as well as emphasising the risk of contamination of RTE foods by *L. monocytogenes* [329].

As remarked by Lundén and colleagues [331], dairy products were incriminated as vehicles in nearly half of the reported listeriosis outbreaks in Europe, while Rietberg and colleagues [153] emphasised that listeriosis is the third-leading cause of food-borne related deaths in the USA. It can be deduced from Table 5 that the USA has experienced several episodes of outbreaks; consequently, the country has embraced a “zero tolerance” level for all RTE foods, irrespective of the risk profiles of the foods. Nevertheless, several other countries are guided by a microbiological tolerance threshold of 100 cfu/g of *L. monocytogenes* in low-risk foods that do not encourage the growth of the bacterium [247]. With respect to Table 5, highlighting the different listeriosis outbreaks in the different countries of the world, the world’s most severe, largest, significant, and deadliest listeriosis outbreak recorded in history to date took place in South Africa in 2018, wherein 937 persons were infected, but 216 cases ended in mortality [247]. According to Smith and colleagues [332], alongside Thomas and coauthors [272], the listeriosis outbreak that resulted in 27% case-fatality rate was linked to the consumption of contaminated polony (RTE meat product), as *L. monocytogenes* sequence type ST6 was recovered from food samples as well as the environment of the facility engaged in the production of the products.

Overall, during the last decade, a global increase in *L. monocytogenes* infections has been observed and attributed to multiple factors [333,334], including the following:

1. Improvement of the diagnostic methods and increased surveillance in public health.
2. The generalisation of food preservation methods, including refrigeration, which permit the growth of *L. monocytogenes*.
3. Industrial development in food generation and the resulting risk associated with large distribution of contaminated food.
4. An increase in the population of susceptible individuals, including the elderly and the immunosuppressed.
5. The rising consumption level of preservative-free RTE foods.
6. The use of antacids and medications that suppress the secretion of gastric acid.

### 4.3. Transmission of L. monocytogenes (Contamination)

**Figure 3:**
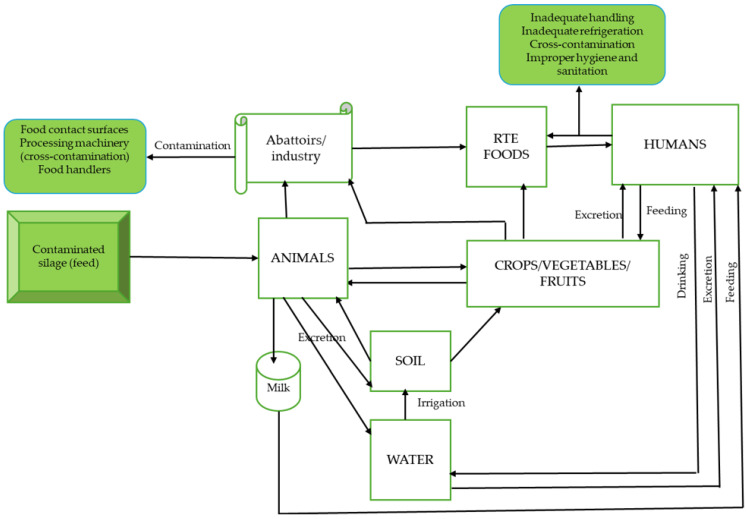
Showing the different channels through which contamination and transmission of *L. monocytogenes* can occur at the food–human–animal–environment interface.

One health approach constituting human–animal–environment (plants and foods) states that the health of one of these components ultimately affects the outcome/health of the other two. Khan and colleagues [237] remarked that there are several sources and routes via which transmission of or contamination with *L. monocytogenes* can occur. As presented in Figure 3 below, the channels include food and food-processing facilities, humans, animals, and environmental sources (water and soil). In this light, *L. monocytogenes* recovered from either food or humans or animals has the potential of being transmitted through the said medium to their counterparts.

#### 4.3.1. Environmental Sources

*L. monocytogenes* is predominantly a ubiquitous environmental saprophyte, with a widespread distribution, occurring in varied environmental niches, including soil, water, vegetation (vegetables, crops/plants, fruits) as well as feed. The prevalence of *L. monocytogenes* in different areas, including water, soil, sewage, feed, surfaces, and farm environments, has been registered [271]. The farm environment is viewed as a very important natural source of raw material contamination because of its regular contamination by the organism [237]. Apparently, based on animal hosts, farm animals are the most affected [150] and the animal feed, surfaces, water troughs, beddings, and feed bunks that are in close contact could become contaminated too. Therefore, in the farm environment, the contaminated feed, water, or pasture could present as possible routes of transmission of *L. monocytogenes* to the animal host [271]. Harrand and colleagues [335] registered a high prevalence of *L. monocytogenes* in the surroundings, including the soil and irrigation water. This could be traced to farm animals, since it is known that they harbour the bacterium in their gastrointestinal tracts; therefore, it can be excreted alongside the faeces into the environment.

Nightingale et al. [336] and Dalzini and colleagues [337] pointed out the influence of seasons on the prevalence of *L. monocytogenes* in farms and their environment, explaining that the bacterium is more prevalent during the spring and winter seasons, unlike in fall or summer. The presence of unidentified asymptomatic shedders encourages the persistence and spread of the bacterium in the environment. Orsi and colleagues [338] explained that *L. monocytogenes* could be shed in faeces released by diseased, recovered, or asymptomatic animals that could lead to contamination of the environment. Equally, the presence of decaying plant matter, moist soil, and water bodies helps the survival of the bacterial species. Subsequently, the faeces have become a source of food- and feed-borne illness in humans and animals as the food production chains represent a continuum from the farm environment to the vulnerable human populations that could be infected with listeriosis [339]. Moreover, the authors remarked that humans and animals can exist as asymptomatic carriers of *L. monocytogenes*, releasing it together with faeces, leading to contamination of the water supply and the soil [159]. The occurrence of contaminated faeces in the environment can lead to an increased level of intraherd transmission, non-deliberate spread to other herds, together with a high risk of human infection [338]. Equally, the application of biological amendments, including treated and untreated/raw manure, is especially useful in organic operations, where the use of synthetic fertilisers is prohibited, but the focus is on health, ecology, fairness, and care principles added to sustainability practices [340]. Similarly, this holds through farms with inadequate waste management systems; the direct application of untreated animal manure on agricultural lands is the traditional management approach [341]. This is an age-long practice as people believe animal waste can serve as a fertiliser, promoting crop growth, added to the fact that their resources are renewable, low cost, easily accessible, and widely available. This practice is extensively associated with shortcomings, causing water, air, and soil pollution [159]. This describes the possibility of faecal-oral route transmission in animals, permitting the maintenance and persistence of the bacterium in the agricultural farm environment [227]. Furthermore, the authors mentioned that the asymptomatic animal carriers represent the channel through which the bacterium is spread in the food industry contaminating milk and meat. Listeriosis occurring in farm animals appears as the major channel of increasing risk of transmission to humans.

#### 4.3.2. Food-Processing Facility/Environment

Unfavourable pathogenic microbes enter the food chain from water, soil, air, and contact with unhygienic equipment, including humans [342]. The natural and wide distribution of *L. monocytogenes* across varied environments and hosts presents an extensive opportunity for transmission to food [340]. Nevertheless, contaminated ready-to-eat foods are regarded as the primary transmission vehicle for listeriosis in humans [269]. Linke et al. [5] purported that the soil and water represent the two principal routes for the transmission of bacteria to plant material, feed, animals, and the food chain. In addition, the potential of contamination and persistence in the processing environment is directly proportional to the level/concentration of *L. monocytogenes* in the raw material subjected to the processing procedure [343]. As previously mentioned, *L. monocytogenes* can survive and thrive under extreme environmental conditions, influencing its spread to a wide range of foodstuffs. The ability to form biofilms on equipment, contact surfaces, and floors, as well as its resistance to disinfectants/sanitisers, facilitate the organism’s persistence in the food production facility and environment [271]. Apparently, the bacterium was detected in several sites in the food facility, including bulk tanks, collectors, filters, and other utensils. It is described as a food-borne pathogen as several listeriosis outbreaks in different countries were associated with a host of foods, including pasteurised and unpasteurised cheeses, crabmeat, smoked salmon, pasteurised milk, ice creams, butter, hotdogs, etc. [271]. In detail, as a ubiquitous and food-borne pathogen, the organism is subjected to numerous physical and chemical stresses that can hinder its growth and survival along the food value chain. The growth of *L. monocytogenes* is one of the most important factors that affect the risk of human listeriosis following the consumption of RTE. The intricacy attached to the growth of the bacterium is that, unlike other food-borne pathogens, it grows at refrigeration temperature and in foods with relatively low moisture content and high salt concentration. Therefore, having the potential to persist and multiply in the food environment makes its control difficult [342].

The processing of RTE food involves many phases including the addition of flavourings, preservatives, emulsifiers, and binders, decontamination, heating, curing, fermentation, or drying. Most of these phases can lead to the reduction in bacterial load in the RTE food at the point of consumption, depending on the effectiveness of the methods, which in turn is influenced by the type of food and the design of the process. However, recontamination of the RTE food can occur due to subjection to further processing and handling. Accordingly, an increased level of handling after heat processing can obviously lead to contamination, as opened packaged RTE foods at homes or found in retail stores are vulnerable to contamination due to inappropriate handling by consumers or customers in the retail shops, as well as inappropriate storage at home or in refrigerators over extended periods [342]. This is very prominent because of the ability of the organism to form biofilms in food and/or the environment. The growth of *L. monocytogenes* in food or the environment can be strain-dependent [188]; this will affect the prevalence of the organism, having a direct impact on contamination and listeriosis development.

Certain factors are described that influence the growth of *L. monocytogenes* in RTE foods, including the characteristics of the product, microstructure of the food matrix, storage temperature and time, the lag phase of the bacterial strain, as well as the presence of competitive microflora [344]. However, as a food-borne pathogen, *L. monocytogenes* responds to such exposures by developing mechanisms that adjust cellular processes to a level that sustains its viability and growth under stated stressful conditions. These mechanisms are known as stress-adaptive responses, which constitute a developmental process originating from the sensing of alterations in the environments and the reprogramming of gene expression to produce stress response proteins to assist the bacteria to survive under the prevailing conditions [345]. Accordingly, several studies demonstrated that a variety of RTE foods of animal origin can act as vehicles for transmission of the said pathogen to consumers [90,268]. Accordingly, Ricci and colleagues [343], under the EFSA panel on biological hazards, outline several potential factors driving the contamination of RTE food and listeriosis in the food chain. These include the following:

1. *The food*
2. *Level of L. monocytogenes contamination in the RTE at retail*
3. *Prevalence of L. monocytogenes in RTE food at retail*
4. *Size of the vulnerable/susceptible population*
5. *Conditions of storage after retail*
6. *Level of consumption*
7. *Virulence of the infecting L. monocytogenes strain*
8. *National surveillance system*

Generally, the increased demand, availability, and extended shelf life of RTE food offers a great likelihood for *L. monocytogenes* to inhabit foods; however, the prevalence of the bacterium is usually high in foods subjected to minimal processing or those vulnerable to contamination following thermal treatment [237].

#### 4.3.3. Humans

They can be viewed as a consumer population and/or food handlers (employees) working at the food-processing facility. These humans can act as vehicles for the transmission of *L. monocytogenes*, functioning in several ways, including asymptomatic carriers, vertical transmission, and contact with infected animals [339]. Firstly, humans become infected by consuming contaminated foods. Asymptomatic carriers appear as a challenge to food safety and asymptomatic colonisation of humans with *L. monocytogenes* may end up in the contamination of food directly or the contamination of the food-processing environment because of inadequate hand hygiene. The accidental or deliberate release of human faeces harbouring the bacterium into the environment can lead to the contamination of plants, water bodies, and other humans and might eventually end up in the food chain. Vertical transmission describe the transfer of the bacterial pathogen from mother to offspring (child) via contact with infected/contaminated animals or hospital-acquired infections [231]. Domestic animals become infected via the ingestion of contaminated water and feed (silage) [346]. Venereal transmission or inhalation are the other possibilities through which animals can be infected. These infected animals can release *L. monocytogenes* in milk, urine, faeces, and other discharges (uterine and nasal) [347]. Therefore, humans can be infected with the organism via direct or indirect contact with infected animals, which in turn can become a source of transmission [348]. Nkhebenyane and Lues [349] opined that the contamination rate is greatly influenced by the ability of the food handlers to respect the hygienic measures during processing and the degree of cross-contamination.

## 5. Prevention and Control of *Listeria* Monocytogenes

*L. monocytogenes* is continuously being ranked as one of the deadliest food-borne pathogens by the surveillance systems of many countries; regardless of its low prevalence, it is associated with a high mortality rate [227]. The organism is a globally distributed bacterial species, invading a wide range of hosts, including food animals; therefore, there are causes for concern not only regarding public health and food safety. However, it is also a vital cause of economic losses in situations where livestock are infected alongside their offspring [350]. Moreover, the bacterium is said to display a biphasic lifestyle attributed to its high level of resilience and versatility that permits it to thrive as a saprophyte in several habitats in the environment and suddenly switch into a dangerous, opportunistic, and intracellular pathogen once it encounters a host [351]. Therefore, seeking ways to prevent and control the said organism in our society is very crucial. Equally, the severity of the listeriosis outbreaks underscores the need for effective preventive control strategies to reduce, control, and/or eliminate *L. monocytogenes*. According to the farm-to-fork and One Health concepts, there is a need to understand the pathogenesis of listeriosis and its epidemiology at the interface of the environment–animals and humans. Since listeriosis is a known food-borne infection caused by an environmental bacterial pathogen, the control of listeriosis in humans should be centred on decreasing contamination along the food chain by *L. monocytogenes* [228]. Therefore, monitoring of the environment in food-processing facilities to enable the identification of niches harbouring the bacterium, followed by upgraded sanitation efforts leading to the eradication of *L. monocytogenes*, is viewed as a cornerstone in bacterial control [228]. The implementation of the environmental sampling programme in the food-processing environment over time is a vital step in the control of *L. monocytogenes*. The effectiveness of this control strategy depends on its design and the company’s response to the positive findings through the sampling programme.

Furthermore, the enhancement of practices in the farm-to-fork continuum on a continual basis and tailored to reduce the entry of the organism into the food chain is shown to represent the core of listeriosis prevention and the methods vital to its control [352]. Extensive efforts have been made through food safety regulations and educational programmes, and by the food industry by way of measures of control and risk analysis models pertaining to *Listeria* contamination from farm to fork. Risk analysis includes risk assessment, risk management, and risk communication. Microbiological risk assessment is an objective procedure comprising four major phases, including hazard identification, hazard characterisation, exposure assessment, and risk characterisation [353]. Swaminathan and Gerner-Smidt [352] recounted that great decreases were observed in the incidence of sporadic listeriosis, ascribing such results to the efforts carried out by food processors and food regulatory agencies while targeting the control of the bacterium. Furthermore, sensitisation and education campaigns of risk groups and consumers with clear information about listeriosis to avoid certain foods described as high-risk foods will go a long way to reduce mortality and morbidity caused by listeriosis [354].

For surveillance and control of *L. monocytogenes* to become efficient, it is imperative to map and comprehend the distribution and the pathogenic ability of the strains that are frequently isolated, considering the high capacity of the organism to adapt to diverse environments, as well as its usefulness in public and animal health worldwide [227]. It is obvious that listeriosis cannot be eliminated, since the causative agent *L. monocytogenes* is said to be ubiquitous, occurring naturally in the environment, thus emphasizing a huge likelihood of contamination of the food we eat from time to time. In addition, the scarcity of simple techniques to identify or detect the presence of contamination with *L. monocytogenes* in the environment, added to a deficiency in knowledge about the risk factors other than silage, are the plausible ways through which the control of listeriosis becomes difficult [231]. Moreover, the management approaches are fraught with challenges caused by the organism’s capability to form biofilms and demonstrate resistance to low temperatures and high salt levels, leading to failure [45]. Similarly, the huge diversity expressed by the genotypes and phenotypes of *L. monocytogenes* isolates added to their weak clonality and adaptability creates difficulties in controlling and managing the organism [355].

The treatment of listeriosis is viewed as a means of control. Beta lactams (amoxycillin, penicillin, ampicillins) are the first choice of drugs, as well as trimethoprim and sulfonamide, in eradicating intracellular listeriosis, as the drugs exert bacteriostatic activity. In addition, combination therapies including cotrimoxazole (trimethoprim-sulfonamide) and beta-lactam-aminoglycoside exhibit bactericidal activity against listeriosis [228]. Shamloo and colleagues [68] explained that when food materials are contaminated with the bacterium, an increase in antibiotic resistance can occur as the bacterium becomes exposed to preservatives, stress conditions, and antibiotics. This is a dilemma because antibiotic resistance is a public health threat on a global level; therefore, ways to control contamination and subsequent transmission within and between niches, animals, and humans require special attention to lessen the incidence of listeriosis. Antibiotic resistance is viewed as a virulence factor, provoking infection. A surveillance programme on antimicrobial resistance in food pathogens is paramount to the control of food contamination, as this will help to prevent the occurrence of the disease, leading to better management of the infection. Even when the disease is manifested in animals, prevention and control could be implemented by adequate disposal of contaminated materials, bedding, and litter, incineration of infected carcasses, and the wearing of proper clothing while manipulating aborted material, retained placenta, and infected animals [231].

Nevertheless, two major steps in minimising listeriosis entail preventing contamination and controlling the incidence of the pathogen in the food chain and foodstuffs. The control measures consist of systematic microbiological monitoring of raw and processed foods in conjunction with sanitation schemes following the detection of the bacterium, complemented by training programmes based on food hygiene for employees (food handlers) [354]. More elaborately, improved hygiene and sanitation involves proper washing of kitchen utensils and equipment at the food-processing facilities and proper washing and disinfection of hands during food production. Hence, contamination must be addressed to reduce the growth of the organism [237]. Considering that the infection is primarily transmitted through foods in humans and animals, good hygiene practices can curb its spread [267].

Furthermore, in circumstances where an outbreak has already occurred, there is a dire need of early recognition of the outbreak via molecular typing, to identify geographically diffused clusters of cases if the subtyping is performed immediately, following the recovery of clinical isolates through standardised procedures [352,356]. The subtype patterns are submitted without any waste of time to a national database. The national database is monitored continuously for cluster detection. This practice is conducted in France, and the UK and has heightened recognition potential in both countries regarding the outbreak of listeriosis. Regardless of the advances, the best approach to protecting public health is still prevention [109].

### 5.1. Emerging Control Technologies

Yousefi and coauthors [357] emphasised that the provision of healthy and safe foods by food industries is one of the most challenging issues, owing to the presence of pathogenic and spoilage microorganisms. The demands of consumers for foods of high convenience, possessing superior characteristics and high nutritive value, that are easy to eat, safe, and with minimum processing, and have longer shelf lives and mouth wearing taste have increased [358]. However, these qualities are impossible to achieve via the use of the present thermal treatments, which results in foods having reduced nutritional and sensory characteristics along with a shorter shelf life [359]. In contrast, non-thermal treatments produce foods with high nutritional and sensory qualities, with a longer shelf life. Accordingly, Jadhav and Choudhary [360] remarked that non-thermal treatment methods [e.g., ultraviolet, pulsed electric field, high pressure processing, cold plasma, ultrasonication, supercritical carbon dioxide and supercritical technologies (pulsed ultraviolet, irradiation)] that are eco-friendly, energy efficient, cost effective, and which do not operate and process foods at higher temperatures for longer period are very eminent. These treatment methods occur among the food-processing techniques that transform raw materials into final product but also make sure that pathogenic bacteria, toxins and other harmful constituents are inactivated [361]. The different non-thermal treatment methods describe treatments approaches wherein processed foods and raw foods, among others, are subjected to the environmental temperature over a short period of time to reduce microbial load and extend the food shelf life, in particular [362]. Their operations often result in minimal use of energy, saving lots of energy and time. However, Bahrami and colleagues [363] highlighted that the composition and characteristics of foods, conditions of processing, and the resistance of *L. monocytogenes* (species dependent) to the processing are among the varying factors that affect the efficiency of these novel strategies. Each technology presents with advantages as well as specific challenges [364].

In the food-processing environment *L. monocytogenes* occurs in multi-species biofilms. In this light, several methods, including probiotics, cold atmospheric plasma, photodynamic inactivation, natural products, chemical disinfectants, antibiofilm materials, nanomaterials, acid electrolysed water, antibiofilm enzymes, quorum sensing inhibitors, and bacteriophages, could be employed to prevent and control *L. monocytogenes*. The treatment methods can be applied as standalone or in sequential order or in combination in the quest to achieve maximum destruction of the microorganisms and increase the shelf life of the foods while retaining their organoleptic, nutritional and textural qualities [365]. Yin et al. [366] emphasised that a combination of the different non-thermal methods offers better effects for controlling biofilms, particularly multi-species biofilms. Jadhav and Choudhary [360] remarked that using a combination of these treatments results in synergistic effects, which help to deal with the limitations associated with individual techniques. Based on the type of food and the purpose of operation or the desired effect, the non-thermal treatments are selected, and they may be described as specific or non-specific, although all these methods destroy cell membranes and genetic materials thus decreasing the microbial load in the food and causing disorganisation of catabolic and anabolic activities in microorganisms [367].

#### 5.1.1. Phage Therapy

Phages are also termed bacteriophages, which are bacterial viruses that infect and replicate only inside a bacterial host. They are almost 50 times smaller than bacteria, occurring in the soil, water and in food products [368]. Song et al. [369] therefore described bacteriophages as natural viral predators of bacteria. In terms of size and morphology, phages can be grouped into various classes, including filamentous and pleomorphic phages, among others, yet can be divided into two types: the lytic (virulent) and the lysogenic phages (temperate) [370] based on their life cycle. The lytic phages are the targeted phage type used to screen against vulnerable food-borne pathogens of interest, including *L. monocytogenes*, thus acting as a tool for biocontrol. They are denoted as lytic because they explore the lytic life cycle strategy, describing a scenario in which the phage adheres to a particular target bacterial strain and injects its genome into the cytoplasm of the host cell. Subsequently, it uses the ribosomes of the host’s machinery to produce its proteins or other enzymes or cell components that are assembled into several copies of the original phage. Following the lysis of the cell at the end of the lytic infection cycle, progeny phage particles arise from the cells and are released to infect another cell [371]. The outcome of the lytic cycle causes a reduction in the viable cell count, and it is described as bactericidal [368]. Accordingly, Liu et al. [372] described bacteriophages as natural, highly specific, and potential candidates to eradicate *L. monocytogenes* without any toxic effects, meaning they are unharmful to consumers. Alternatively, in the lysogenic replication cycle, attachment to the host cell followed by introduction of its genome into the cytoplasm of the host cell occurs like in the lytic cycle. However, in this instance, the genome of the phage is integrated into the chromosome of the bacterial cell or exists as an episomal element. Then, the genome is replicated and transferred into daughter bacterial cells known as prophages without killing them [371].

Phages demonstrate a high degree of selectivity when infecting the bacterial host, i.e., they can infect only a single bacterial species or even specific strains of a species [373]. It is quite interesting, as the phage should not infect other species, which will eventually lead to the death of non-pathogenic commensal bacteria, in so doing diluting the optimum dosage for the targeted pathogen. Therefore, the phage chosen for antibiotic therapy should, specifically, target the pathogen [374]. Several critical features are considered for a phage to be considered as a good candidate, and these factors are observed to affect the effectiveness of the phage. These include that the phage must be lytic, polyvalent, thermostable, and lack microbial resistance and virulence genes [368]. Other factors that can influence the interaction between phages and their hosts, thereby affecting the effectiveness of phages, entail ionic strength, pH, nature of food (whether liquid or solid), cell wall and cell membrane penetration, interference of phage particle diffusion by the presence of other substances, receptor recognition, and binding, as well as the process involved in virus genome internalisation [262]. Having such properties of not influencing microflora other than the targeted ones and not influencing the sensory qualities of the final product, bacteriophages are regarded as a great weapon against pathogens in food production [262]. Adebesin and colleagues [249] noted that the multifaceted applications of phages for the detection and biocontrol of pathogens underlines the potential of phages in increasing food safety and clinical diagnostics.

In addition, Hagens and Loessner [375] registered a total number of phages beyond 500 that are specifically infecting *Listeria* species, the majority of which, however, are temperate; therefore, most of them are not useful in inhibiting *L. monocytogenes* in food products and the food-processing plants. Consequently, lytic bacteriophages are vital tools in the biocontrol of *L. monocytogenes*; however, they are endowed with distinct properties and, therefore, their selection in turn influences the pathogen inhibition activity [262]. Gutiérrez et al. [376] through a comparative analysis noted a difference in the inactivation potential of two phage-based products (Listex^TM^ P100 and ListShield^TM^) in Spanish dry-cured ham and food contact surfaces. The former phage treatment caused *L. monocytogenes* cells to be reduced below the detection limit (<10 cfu/cm^2^) after 24 h at 4 and 12 °C while ListShield^TM^ demonstrated a 3.5 log reduction in *L. monocytogenes* only in samples with a high level of contamination (10^5^ cfu/cm^2^) after 14 days incubation at 4 °C. Table 6 harbours some examples of *Listeria* phages and their descriptions.

Currently, *Listeria* phages display a certain degree of serotype specificity based on the presence or the lack of sugars, rhamnose, and N-acetylglucosamine of wall teichoic acids that serve as binding receptors during the adsorption phase of the infection cycle [381]. Therefore, the serotypes of *L. monocytogenes* vary owing to the presence or absence of these sugars [380]. Song et al. [369] registered that *L. monocytogenes* can be divided into at least 13 serotypes based on cell surface antigenic determinants. However, only the serotypes 1/2a and 4b are the most recovered from food and environmental samples [382]. Interestingly, Guenther et al. [383] explained that the efficacy of a phage is largely affected by the structure of the food matrix. Stones and colleagues [384] performed the treatment of different food matrices (artificially contaminated milk and baby spinach) stored at 8 °C and 12 °C and revealed a significant reduction in the numbers of *L. monocytogenes* over the shelf life. The authors further noted that the phage P61 was virulent with its lytic spectrum spanning over serotypes 1/2a, 1/2b, 1/2c,4b, 4e, and 6a. In another study, Ribeiro et al. [385] attempted to reduce *L. monocytogenes* by treating both pasteurised milk and broth media using Listex P100 while varying the temperature and duration of storage of the milk. The authors found that bacterial counts were reduced in the following ranges, 1.5–3.5 log cfu/mL and 2.5–4.4 log cfu/mL in milk and broth media, respectively, in correlation to the conditions of storage. Following published findings based on the effectiveness of phage-based treatment of dairy products in inhibiting *L. monocytogenes*, El-Bakry and colleagues [386] emphasised that dairy products (e.g., raw milk, pasteurised milk, cheese, yoghurt, butter, and cream) are of complex matrices harbouring microstructures that differ depending on how the milk is being processed and stored. These features may in turn influence the interaction between the phage and bacterial cells. Nevertheless, Grigore-Gurgu et al. [387] opined that bacteriophages can be used in dairy with no risk of changing the starter culture, embodied by the lactic acid bacteria population; however, a striking feature of safety is derived from several studies that pointed at the regrowth of *L. monocytogenes* on cheese treated with phage during prolonged storage, even with an initial reduction in *Listeria* counts [385].

Similarly, Cucić and coauthors [388] in their study degraded the biofilm developed by *L. monocytogenes* ATCC 19111 (serovar 1/2a) through the activity of phages grouped under the genus Pecentumvirus, including the commercially available Listex P100. The authors further registered a 2 log reduction in *L. monocytogenes* 19111 biofilm-producing bacteria caused by the phage CKA 15. In addition, Gόmez-Galindo et al. [389] optimised the use of a commercially available phage-based product (phageGuard Listex^TM^) as a control strategy against *L. monocytogenes* in fresh-cut industries located in Spain and Denmark. The authors demonstrated no impact on sensory qualities (e.g., flavour, visual appearance, texture, browning, spoilage) of RTE shredded iceberg lettuce over the shelf life following the post-treatment process, which involved the application of a fine mist solution of phageGuard Listex^TM^. In the same vein, Schellenkens et al. [390] noted that the *L. monocytogenes* phage P100 showed no interference on the functional lactic acid bacteria in the tested cheese or alteration of product characteristics; furthermore, Perera et al. [391] highlighted that the commercially available cocktail product ListShield^TM^ exerted no effect on the organoleptic qualities (e.g., smell, taste, and sight) of pre-sliced oven-roasted turkey breast, cooked ham, meat bologna, and roast beef. Therefore, the level of reduction in *L. monocytogenes* by *Listeria* phages could be affected by the following factors: the ratio of the bacteriophage titre and the level of contamination (initial concentration), strain divergence of the pathogen, type of food, the contact between the phages and the host, the presence of host resistance to the phages, chemical composition and characteristics of products, physicochemical factors of food, and the storage time and conditions [376,387]. Notwithstanding this, *Listeria* phages provide a safe and environmentally friendly strategy for the decrease in numbers of *L. monocytogenes* cells in certain RTE foods [384].

Generally, challenges encountered in phage therapy are focused on technical issues in phage screening and preparations during research and development for food products in general, and precisely, in fermented meat products with low pH and high salinity, added to the low likelihood of contact between the phage and the bacterium on the solid surface [392]. Secondly, in circumstances where the present control strategies against a specific food are ineffective, more detailed standard operating procedures tailored against specific pathogen–food combinations are needed prior to their incorporation into the food industry of phages or their cocktails with proven efficacy [368]. Thirdly, there is the issue of sufficient efficacy, as Kocot et al. [393] reported that the efficacy of phages on pathogen reduction is restrained to some extent, appearing in the range of between 0.5 and 2 log reduction. Fourthly, Moye and coauthors [378] raised concerns about the development or emergence of phage-resistant mutants owing to the widespread use of bacteriophage treatments.

#### 5.1.2. High-Pressure Processing (HPP)

This is a preservation technique also termed high-hydrostatic-pressure processing (HHP) or ultra-high-pressure processing (UHP). It is also known as bridganization [360]. UHP is one among the oldest forms of treatment technologies described as non-thermal which is employed in the food and allied sectors [361]. The instrumentation of UHP is very simple, consisting of a pressure compartment wherein the food is kept, and pressurised by introducing water from the bottom while the lid is closed. Bahrami et al. [363] noted that the behaviour of the food product is altered based on Le Chatelier’s principle because the application of pressure brings about a shift in the equilibrium of the system toward occupying the smallest volumes. On a microscopic scale, at a constant temperature, it increases the degree of ordering molecules in a specified substance. Once the desired pressure is developed, it is maintained throughout the operation process, occurring between 250 and 600 MPa for a certain period (usually for a few minutes) [394]. The application of a uniform pressure throughout the process is a distinctive advantage over the thermal process, because it is unaffected by the size, shape, or composition of the food [364]. Listed amongst the additional factors influencing the inactivation of *L. monocytogenes* via HPP include the applied pressure, temperature, and holding time (these are called process parameters) [395]. Roobab and colleagues [396] employed HPP on seafood products that led to the effective reduction in microbial load, including *Listeria monocytogenes* and *Vibrio parahaemolyticus*, which are usually linked with seafood-borne illnesses. Such high pressures result in a high reduction in microbial load, caused by the breaking of secondary and quaternary structures of proteins, resulting in the denaturation of proteins that lead to cell death and alteration in the pH of microbial cells with disorganisation of structures of the cell, thereby causing cell death. Roobab et al. [396] emphasised that high pressure influences the integrity of the cell membrane of the microorganisms and restrains the activities of necessary enzymes, thereby preventing the growth and proliferation of microbial cells. Furthermore, Georget et al. [397] reported changes in the fluidity of the cell membrane and loss of intracellular pH, leading to the disruption of cells, which are the consequences of the inactivation of *L. monocytogenes* cells. Also, pressure may result in the dissociation of the microbial ribosomes and limit the viability of cells, therefore extending the shelf life and improving the safety of the food [364]. Kulawik et al. [398] reported that further extension of the shelf life of the seafood products could be reached via employing HPP which resulted in the inactivation of enzymes responsible for food spoilage. Meanwhile, the natural colour, the appearance and organoleptic characteristics as well, as the flavour of the food being treated remain unchanged or intact [397]. However, the high investment cost associated with HPP technology stands as one of the major barriers to its implementation, especially for small and medium-sized enterprises [399]. Similarly, Aganovic et al. [400] noted that based on the conditions of the HPP process, the death of cells may not occur, but only sublethal damage from which the pathogen may recover through the repairs of the injuries during storage. Therefore, HPP might not consistently meet the 5 log reduction in pathogen bacteria stipulated by the FDA, particularly in RTE foods.

#### 5.1.3. Antimicrobial Active Packaging

Zhao et al. [401] indicated that contamination by microorganisms of RTE meat occurs at the surface, so then the application of natural antimicrobials in packaging films (antimicrobial packaging) can aid in the control of pathogenic and spoilage microorganisms on the product. There exist several biological agents or substances or compounds with antilisterial properties that can be incorporated into active packaging, which can be employed in the control (reduce or eliminate) of *L. monocytogenes* in food products, ensuring the safety of the products. Antimicrobial packaging is a form of active packaging that permits the release of an antimicrobial substance to suppress the activities of specific microorganisms via extending their lag phase, thus leading to the improved quality and safety of food during an extended period of storage. Fadiji et al. [402] mentioned that antimicrobial packaging may exist in different forms, including antimicrobial sachets inside packaging, packaging films, and coatings incorporated with active antimicrobial agents. Depending on a particular product, the choice of the best packaging system is influenced by the nature of the product, the desired shelf life, and the requirements needed for storage, added to legal considerations.

The prevention and control of the growth of *L. monocytogenes* in food involves bacteriophages, lactic acid-producing bacteria (competitive bacterial species), bacteriocins, metals, chemicals, essential oils, and endolysins and their derivatives, which are the biological agents available [387]. These antimicrobial agents are expected to occur on the surface of the food above their minimum inhibitory concentrations for them to be effective [402]. Endolysins, also known as enzybiotics, are peptidoglycan hydrolases produced by phages at the last stage of the lytic cycle. Recently, lactic acid-producing bacteria have been viewed as an alternative approach to chemical preservatives in the food industry to ensure food safety via the expression of antagonistic effects against food-borne pathogens. Their antagonistic effect is ascribed to the production of antimicrobial molecules or substances, namely, bacteriocins, diacetyl, organic acids, hydrogen peroxide, and carbon dioxide [403].

Furthermore, bacteriocins are known antimicrobial peptides produced by lactic acid-producing bacteria, which are categorised into three main classes, I, II, III, and offer a mechanism of defence against competing bacteria and pathogens [387]. Of the three classes of bacteriocins, classes I and II are the most utilised antimicrobial compounds employed in food preservation. Nisin, a lantibiotic, is a small peptide produced by lactic acid-producing bacteria, grouped under the class I bacteriocin, and it was the most examined in relation to biocontrol of *L. monocytogenes* in food. In a study performed by Espitia and colleagues [404], the potential application of cellulosic antimicrobial packaging films for the preservation of bologna and inhibition of *Listeria* biofilm formation was studied. The bacteriocin pediocin was incorporated at different concentrations (30, 40, 50% *w*/*w*) and the experiment tested at 10 and 25 °C. They found that the cellulosic film impregnated with 50% pediocin reduced 1.2 log cycles of *L. monocytogenes* growth on sliced bologna after 9 days and prohibited the formation of biofilm on the surface of the packaging and bologna. Nisin stands as the only bacteriocin approved as a food additive (E234) and is employed in over 48 countries in meat, dairy, and vegetable products [405,406].

Furthermore, Burt [407] described essential oils as hydrophobic aromatic oils produced by plants as secondary metabolites to shield themselves from pests. Essential oils, constituting terpenoids, ketonic bodies, aldehydes, and phenols occur among the different classes of bioactive substances, demonstrating strong antimicrobial properties with great potential to prevent the growth of diarrheagenic bacteria (undesired bacteria in food), decrease inflammation of the intestines, and regulate bowel activities [249]. Bahrami et al. [408] stated that free or encapsulated forms of natural antimicrobial compounds can be used singly or in combination with other technologies to control pathogens in food. Essential oils are natural preservatives employed in the food industry, although applied differently based on the type of food. Khaleque et al. [409] studied the application of crude and commercial essential oils of clove (5 and 10%) and cinnamon (2.5 and 5%) against *L. monocytogenes* in ground beef stored for 7 days at 0 and 8 °C, as well as stored for 60 days at −18 °C. They realised that based on the storage temperature and time, the *L. monocytogenes* population was reduced by 5% commercial cinnamon essential oil in the order 3.55–4 log cfu/g of the ground beef.

Yousefi and colleagues [357] explained the antimicrobial activity of essential oils through various actions, including changing the profile of fatty acids, altering cell membrane structure, increasing the permeability of cells, and inhibiting the functional properties of the cell wall, and affecting the proteins located in the membrane. The effectiveness of essential oils depends on the type of oil, its concentration, and compatibility with the food matrices. However, the application of essential oils as food ingredients is fraught with challenges, including altering the organoleptic characteristics of the food due to their intense aroma, thereby rendering them unacceptable to consumers. This is because the essential oils affect the activity of lactic acid bacteria and the sensory properties of the food, e.g., cheese [387]. To address this mishap, food processors resorted to the use of essential oils at low concentration, which resulted in decreased antimicrobial action or inefficiency caused by the possible interaction between the essential oils and fats, carbohydrates, or proteins found in the food, added to external factors, including light, oxidation, or heating [410]. The authors hereby suggested the application of essential oils in the active packaging of food products as one of the effective strategies to maximise the use of these substances, explaining that the active compounds migrate from the packages into the foods, rendering protection against microbes.

In addition, Kawacka et al. [262] indicated that the immobilisation of bacteriophages on modified cellulose membranes via electrostatic interactions, because of the charge difference on the external structure of the phage in the form of active packages, has emerged as an option for its application to food. Immobilised *Listeria* phages were applied to RTE meats packaged under vacuum or modified-atmosphere packaging and resulted in decreased growth of *L. monocytogenes* to less than a 0.5 log cycle through the shelf lives of the RTE food [411]. The findings of a study performed on pre-cooked turkey breast revealed a 99.99% (4 log) reduction in the growth of *L. monocytogenes* cells that were in contact with *Listeria* phages immobilised to Xanthan coatings applied on polylactic acid (PLA) films, relative to xanthan coated PLA or uncoated PLA alone [412]. However, the authors considered other findings alongside theirs and suggested the combination of phage therapy and other antimicrobial compounds, as opposed to its application as a standalone, to be a better strategy to address *L. monocytogenes* contamination in food. Furthermore, Kawacka et al. [262] highlighted the regrowth of *L. monocytogenes* following the application of phage therapy during further storage even if the organism was initially reduced.

Grigore-Gurgu et al. [387] mentioned that combining selected lactic acid-producing bacterial strains with various antimicrobial compounds (e.g., bacteriocins) into polymeric films and their subsequent immobilisation on edible coatings to develop active packaging is presented as a future innovative approach to eliminate the risk of *L. monocytogenes*. Combination treatment usually demonstrates additive or synergistic actions or effects of the different substances involved to trigger a better action, thereby enlarging the spectrum of the microbial targets, which can lead to a greater reduction in the pathogen [262]; the use of bacteriocin-producing lactic acid bacteria would demonstrate an advantage over the application of only bacteriocins, which are easily degraded.

## 6. Conclusions

*L. monocytogenes* is described as an intracellular, opportunistic, and food-borne pathogen infecting animals, birds, and humans, causing listeriosis, which is of global public health concern because of its greatest fatality rate of 20–30% compared to other pathogens originating from food [413]. The organism thrives and grows in the environment, with the possibility of contaminating different varieties of food and food-producing facilities [109]. Therefore, it is a key economic burden on the food industry regarding costs of analysis and potential product recalls. It can contaminate food products during or after processing, posing a significant threat to the food industry, especially ready-to-eat food, owing to its tendency to grow and thrive over a broad range of adverse environmental conditions. The high-risk foods linked with listeriosis consist of the RTE of both animal and plant origins, along with those foods that are refrigerated yet are not subjected to any extensive heat treatment prior to their consumption [414]. Based on the drastic rise in the consumption of longer shelf life RTE foods, less preserved food, and processed food, the prevalence of *L. monocytogenes* in the said fraction of food is crucial to evaluating the likelihood of spread and risk posed to the end users [122]. Several parameters entailing the ability to grow at cold temperatures, the favourable conditions at the retail level and in the course of transportation, as well as home storage, facilitate this pathogen to attain a hazardous concentration before consumption. Consequently, control via monitoring of the food-processing environments is very pertinent. Generally, the control of *L. monocytogenes* in the food-processing environments is targeted at reducing or eliminating the peril of recontamination due to the presence of biofilms on industrial surfaces. Therefore, every individual contamination vector must be identified, and control measures instituted to eradicate the bacterium at each point of entry, including drains, floors, walls, ceilings, processing equipment, refrigeration units, air/particle sources, etc. Owing to the distinctive features and characteristics of *L. monocytogenes*, great efforts involving a combination of two treatments, whether physical or chemical, are needed to control the bacterium at levels that avoid food contamination [48].

Antibiotics are of great relevance in the treatment regimen involving listeriosis; however, the effectiveness of these drugs is reduced over time owing to the rising level of antibiotic resistance displayed by *L. monocytogenes*. Based on the rampant use and abuse of antibiotics in communities and farms, the organism exerts an increased level of resistance to conventional antibiotics, leading subsequently to a critical level of MDR development [347]. The organism displays a great diversity in its strains, corresponding to varying levels of resistance to temperature (low and high), acidity (high and low), osmolarity, disinfectants, and antibiotics [45]. The resistance of *L. monocytogenes* to antibiotics has become a threat to the existing effective treatment for an ever-increasing range of microbial infections [67]. It results in decreased efficacy of antibiotics, complications in treatment, time-consuming, more serious, and prolonged infections, and expensive treatments [231]. Therefore, antibiotic and multidrug resistance expressed by the different strains of *L. monocytogenes* is the main threat to global public health, food security, and food advancement, since it causes difficulties in the treatment of diseases, negatively impacting the success of prevention and treatment in both animals and humans, thus rendering antibiotics ineffective [347].

Overall, the varying prevalence rates of *L. monocytogenes* together with their antimicrobial-resistant counterparts from various food sources, warn of a potential future threat to the health of the population. Therefore, increasing the awareness of people relating to listeriosis and the causative agent, *L. monocytogenes*, added to the designing of adequate policies and control measures for the disease, are highly recommended [292,415].

## Figures and Tables

**Table 3:** Genetic evolution in *L. monocytogenes* (adopted from Quereda et al. [110]).

Lineages	Serotypes	Clonal Complexes	Sources	*Listeria* Pathogenicity Islands
I	1/2b,3b,4b,4e	CC1, CC2, CC4, CC6 (hypervirulent)	Clinical isolates of humans	LIPI-1, LIPI-3, LIPI-4
II	1/2a, 1/2c, 3a, 3c	CC7, CC9, CC121 (hypovirulent)	Clinical and food, but more in food	LIPI-1
III	4b, 1/2a, 4a, 4c	Rarely isolated	Predominantly animal sources	LIPI-1, LIPI-4
IV	4a, 4c			LIPI-1, LIPI-4

CC, clonal complex; LIPI, *Listeria* pathogenicity island.

**Table 4:** Some of the key parameters of the pathogenesis of *L. monocytogenes*.

Hosts	Infectious Dose	Symptoms	Disease Outcomes	Examples	References
Healthy population (immunocompetent)	10^7^–10^9^ colony-forming units	Nausea, headache, fever, diarrhoea, vomiting, muscle pain, abdominal pain	Febrile gastroenteritis	Healthy individuals.	Pouillot et al. [233]; Bagatella et al. [227].
Susceptible/vulnerable/high-risk population	10^4^–10^6^ colony-forming units	Encephalitis, meningitis, endocarditis, septicaemia	Invasive listeriosis	Elderly, neonates. HIV/AIDS individuals, patients undergoing chemotherapy.	Heiman et al. [234]; Pérez-Trallero et al. [235]; Lachmann et al. [236].

**Table 6:** Some *Listeria* phages and their descriptions.

*L. monocytogenes* Phages	Descriptions	References
ListShield^TM^ (formerly LMP-102)	Commercially available phage product. A cocktail of six (6) distinct lytic phages as follows: LIST-36 (ATCC#PTA-5376) LMSP-25 (ATCC#PTA-8353) LMTA-34 (ATCC#PTA-8354) LMTA-57(ATCC#PTA-8355) LMTA-94 (ATCC#PTA-8356) LMTA-148 (ATCC#PTA-8357)	Sadekuzzaman et al. [377].
PhageGuard ListexTM (formerly ListexTM P100)	Commercially available product. Only one broad host range, phage P100	Moye et al. [378].
P70	Broad host range infecting *Listeria* species serovars: 1/2a, 1/2b,1/2c,4a,4c,4d,4e5,6a, and 6b with similar efficiencies. It has a distinct virion morphology, genomic size, and structure that is unrelated to any *Listeria* phage so far identified	Schmuki et al. [379].
LP-018	Assigned to *Homburgvirus* genus with the distinct trait to infect phage-resistant mutants	Vongkapamin et al. [380].

## Data Availability

No new data were created or analyzed in this study.
